# The Road to Infection: Host-Microbe Interactions Defining the Pathogenicity of *Streptococcus bovis/Streptococcus equinus* Complex Members

**DOI:** 10.3389/fmicb.2018.00603

**Published:** 2018-04-10

**Authors:** Christoph Jans, Annemarie Boleij

**Affiliations:** ^1^Laboratory of Food Biotechnology, Institute of Food Nutrition and Health, Department of Health Science and Technology, ETH Zurich, Zurich, Switzerland; ^2^Department of Pathology, Radboud Institute for Molecular Life Sciences, Radboudumc, Nijmegen, Netherlands

**Keywords:** *Streptococcus gallolyticus*, *Streptococcus lutetiensis*, *Streptococcus infantarius*, virulence, infective endocarditis, colorectal cancer, pilus, microbiota

## Abstract

The *Streptococcus bovis/Streptococcus equinus* complex (SBSEC) comprises several species inhabiting the animal and human gastrointestinal tract (GIT). They match the pathobiont description, are potential zoonotic agents and technological organisms in fermented foods. SBSEC members are associated with multiple diseases in humans and animals including ruminal acidosis, infective endocarditis (IE) and colorectal cancer (CRC). Therefore, this review aims to re-evaluate adhesion and colonization abilities of SBSEC members of animal, human and food origin paired with genomic and functional host-microbe interaction data on their road from colonization to infection. SBSEC seem to be a marginal population during GIT symbiosis that can proliferate as opportunistic pathogens. Risk factors for human colonization are considered living in rural areas and animal-feces contact. Niche adaptation plays a pivotal role where *Streptococcus gallolyticus* subsp*. gallolyticus* (*SGG*) retained the ability to proliferate in various environments. Other SBSEC members have undergone genome reduction and niche-specific gene gain to yield important commensal, pathobiont and technological species. Selective colonization of CRC tissue is suggested for *SGG*, possibly related to increased adhesion to cancerous cell types featuring enhanced collagen IV accessibility. *SGG* can colonize, proliferate and may shape the tumor microenvironment to their benefit by tumor promotion upon initial neoplasia development. Bacteria cell surface structures including lipotheichoic acids, capsular polysaccharides and pilus loci (*pil1, pil2*, and *pil3)* govern adhesion. Only human blood-derived *SGG* contain complete pilus loci and other disease-associated surface proteins. Rumen or feces-derived *SGG* and other SBSEC members lack or harbor mutated pili. Pili also contribute to binding to fibrinogen upon invasion and translocation of cells from the GIT into the blood system, subsequent immune evasion, human contact system activation and collagen-I-binding on damaged heart valves. Only *SGG* carrying complete pilus loci seem to have highest IE potential in humans with significant links between *SGG* bacteremia/IE and underlying diseases including CRC. Other SBSEC host-microbe combinations might rely on currently unknown mechanisms. Comparative genome data of blood, commensal and food isolates are limited but required to elucidate the role of pili and other virulence factors, understand pathogenicity mechanisms, host specificity and estimate health risks for animals, humans and food alike.

## General introduction to the relevance of the *Streptococcus bovis/Streptococcus equinus* complex

*Streptococcus bovis/Streptococcus equinus* complex (SBSEC) bacteria are Gram-positive species that inhabit the gastrointestinal tract (GIT) of animals and humans. Most SBSEC have been described as commensal bacteria, but some cause serious infections such as bacteremia and infective endocarditis (IE) in humans and animals and match the pathobiont description (Chow et al., 2011; Boleij and Tjalsma, 2013; Jans et al., 2015, 2016). They are associated with underlying conditions including occult colorectal cancer (CRC) (Boleij et al., 2011c), which highlights the importance of SBSEC members in public- and animal health alike.

Furthermore, SBSEC members are detected in food products including fermented milk in sub-Saharan Africa, Asia and Southern Europe, fermented fish in Asia and fermented plants in sub-Saharan Africa and Latin America suggesting a range of habitats and adaptability to different environments for these bacteria (Jans et al., 2015, 2017). Recent advances in phenotypic and molecular technologies provide more detailed classification abilities at various levels from species to sequence type. This advanced classification scheme helps to elucidate the SBSEC population structure, disease associations, transmission routes and host specificity (Dumke et al., 2014; Shibata et al., 2014; Jans et al., 2016). It is still unclear how SBSEC members establish from commensal organisms to pathogens, particularly relating to survival, colonization, adhesion, invasion and interaction with the host immune system. Furthermore, the causality of SBSEC in CRC is not yet proven which leaves the bacterial-driver-passenger model as important theory to describe the potential mechanisms of host-microbe interaction (Tjalsma et al., 2012).

**Figure 1:**
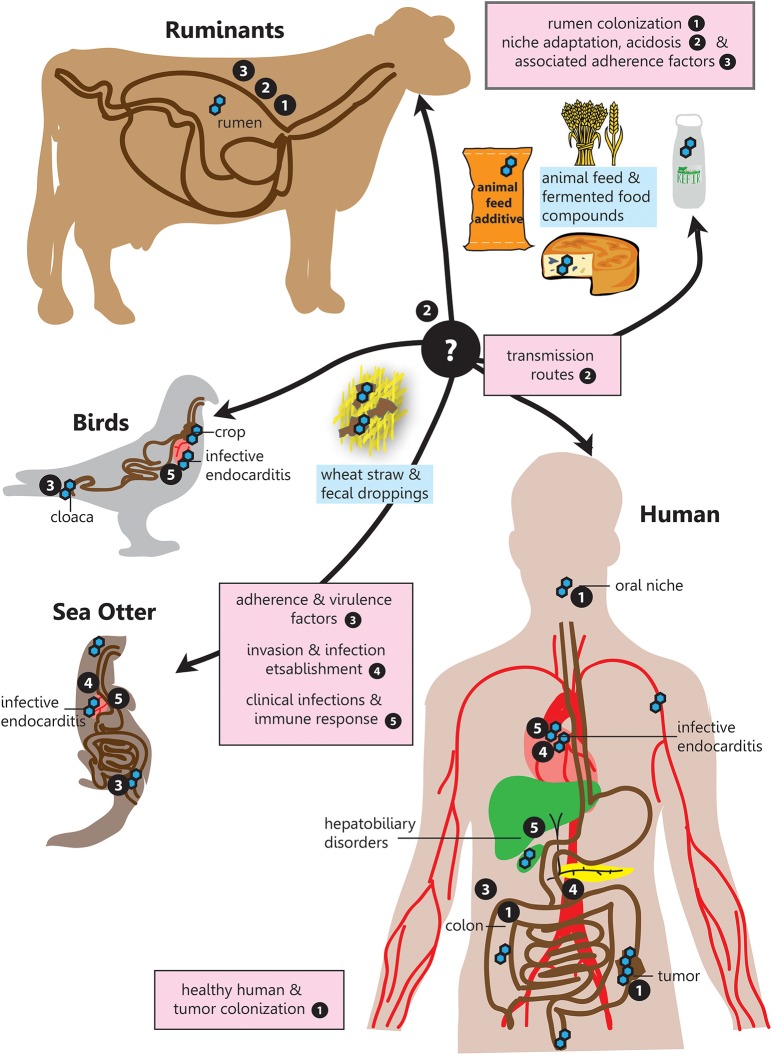
SBSEC - the road to infection. Graphical overview of the different niches inhabited by SBSEC members as well as relevant aspects of host colonization, adherence, invasion and infection covered in the corresponding chapters indicated by bullet point numbers. 1. Prevalence and colonization of SBSEC in animals and humans, 2. Transmission and niche adaptation of SBSEC members, 3. Mechanisms and virulence factors responsible for adhesion and host colonization by SBSEC members, 4. Invasion and infection establishment, 5. Clinical infections and host-immune response due to SBSEC in animals and humans.

Therefore, this narrative review aims to provide a comprehensive overview for the following questions in relation to SBSEC members on their road to infection regarding prevalence, transmission, niche colonization and mechanisms for adhesion, invasion and infection establishment within the human-animal-food system (Figure 1):

1. What abilities help SBSEC members to colonize different body sites or ecological niches and facilitate transmission?
2. Which factors determine SBSEC to evolve from commensal to pathogen?
3. What is the prevalence of SBSEC members in different habitats in relation to clinical manifestations and infections?
4. Which genetic factors are known to encode for these abilities and can be linked to experimental studies?

## Basic taxonomy and identification of the SBSEC

SBSEC members are group D streptococci, although the Lancefield group D antiserum reaction is not ubiquitous (Beck et al., 2008). The SBSEC is comprised of several different species, which in this review will be used according to following nomenclature: *Streptococcus equinus* (*SE*), *Streptococcus infantarius* subsp. *infantarius* (*SII*), *Streptococcus lutetiensis* (*SL*), *Streptococcus alactolyticus* and three subspecies of the clade *Streptococcus gallolyticus*, namely *gallolyticus* (*SGG*), *macedonicus* (*SGM*) and *pasteurianus* (*SGP*). The taxonomic assignment of *SL* as separate species is not fully agreed upon and also referred to as *Streptococcus infantarius* subsp. *coli* (Dekker and Lau, 2016). When no distinction was made between subspecies, the old nomenclature *Streptococcus bovis* (*SB*) was used (Schlegel et al., 2000, 2003; Poyart et al., 2002; Jans et al., 2015). Biotype differentiation is based on the ability to produce acid from mannitol (biotype I = *SGG*) or not (biotype II = *SE, SGP, SL*, and *SII*) (Schlegel et al., 2003; Jans et al., 2015). For full phenotype descriptions, we refer to Bergey's Manual and their implementations in API and VITEK identification approaches (Whiley and Hardie, 2009).

Alternative identification approaches utilize Matrix Assisted Laser Desorption/Ionization Time Of Flight Mass Spectrometry (MALDI-TOF MS) to identify and discriminate species of the SBSEC but results in unreliable identification of *SGM* and a roughly 80% identification rate for *SGG, SGP* and *SL* (Hinse et al., 2011a; Ben-Chetrit et al., 2017). Furthermore, DNA-based approaches are widely applied using single gene PCR and qPCR assays on 16S rRNA gene (Jans et al., 2012b), *sodA* (Poyart et al., 1998), *groES/groEL* (Chen et al., 2008; Lazarovitch et al., 2013; Sheng et al., 2014), *recN* and *gyrB* (Lopes et al., 2014) as well as multi locus sequence typing (Dumke et al., 2014; Shibata et al., 2014; Jans et al., 2016).

## Prevalence and colonization of SBSEC in animals and humans

### Prevalence and colonization in animals

SBSEC are mainly described as colonizers of the rumen, crop and cloaca of animals and colon of humans. SBSEC members have been isolated from the GIT or blood system of birds, companion animals, livestock (ruminants, poultry and pigs), marsupials, aquatic mammals and game (Jans et al., 2015), but prevalence data is limited to birds, cattle and lamb. *SGG* was found in over 90% of fecal droppings in turkey flocks and reached up to 80% prevalence in pigeon crop and cloaca samples (De Herdt et al., 1994a,b; Schulz et al., 2015). SBSEC members were also isolated from chicken crops, but less frequently and not as predominant bacteria (Baele et al., 2001).

In ruminants, SBSEC members are considered as aerotolerant components of the rumen epithelial surface (epimural) microbiota (Mead and Jones, 1981). SBSEC members are estimated to contribute 10^6^ to 10^7^ cells per milliliter of rumen content (Hudson et al., 2000). Their prevalence in cattle is estimated between 20 and 90% with lowest prevalence at early ages (Jans et al., 2015) while in lambs an early live predominance of SBSEC is suggested (Mueller et al., 1984). Colonization and predominance is likely affected by feed composition as shown for reindeers where high SBSEC colonization correlates with higher quantities of starch-rich feed during the summer months (Orpin et al., 1985). SBSEC members are also supplied directly to young calves and goats as probiotics to support the establishment of an anaerobic rumen microbiota and to benefit from consistent α-amylase activity for feed digestion (Kmet et al., 1993; Kumar et al., 2016). SBSEC members therefore seem to form an integral part of the GIT microbiota of birds whereas ruminants feature age-dependent and possible host-associated SBSEC prevalence.

### Prevalence in healthy humans

The fecal carriage rate of SBSEC member in humans is varying from five to over 60% (Table 1) (Klein et al., 1977; Noble, 1978; Potter et al., 1998; Abdulamir et al., 2010; Al-Jashamy et al., 2010; Chirouze et al., 2013; Lopes et al., 2014; Boltin et al., 2015; Dumke et al., 2017; Kaindi et al., 2017). This variation might depend on detection techniques and regional differences. Furthermore, most studies were conducted using a hospital-derived population with possible differences in age, sex and underlying diseases that limits extrapolation to the general population. (Huang et al., 2008; Chirouze et al., 2013; Lopes et al., 2014; Dumke et al., 2017; Harris et al., 2017). Oral niche colonization in humans seems to be infrequent, but isolation of *SII, SGP*, and *SE* was confirmed from dental plaques and root caries lesions (Sissons et al., 1988; Shen et al., 2005; Arul and Palanivelu, 2014).

**Table 1:** SBSEC prevalence in the GIT in studies including healthy people and patients with gastrointestinal conditions sorted by year of publication.

**Country**	**Participant selection**	**Sample type**	**Sample set**	**Sample size**	**SBSEC prevalence (%)**	**Individual species detected**	**Isolation technique**	**Identification technique**	**References**
USA	Patients selected for CRC, inflammatory bowel disease, non-colonic neoplasms, gastrointestinal disorders. Controls among hospital workers and patients with no apparent gastrointestinal disease	Rectal swabs	Controls	105	11	n.d.	Selective culturing	Phenotypic identification	Klein et al., 1977
			CRC patients	63	56				
			IBD	25	28				
			Non-colonic neoplasma	21	19				
			GID	37	14				
UK	Hospital population in- and outpatients	Stool	Adults	39	5	n.d.	Selective culturing	Phenotypic identification	Noble, 1978
			Neonates	21	24				
USA	Outpatients undergoing colonoscopy for suspected CRC and polyps	Stool	Stool before colonoscopy	35	3	n.d.	Selective culturing	Group D streptex	Norfleet and Mitchell, 1993
		Aspirated colon fluid	Aspirated colon fluid	40	8				
		Tissue biopsies	Normal remote	40	0				
			Normal adjacent	40	0				
			Adenoma	33	3				
			Carcinoma	6	0				
UK	CRC cases matched to patients having surgery for benign disease or diagnostic colonoscopy	Stool	Control	23	13	n.d.	Selective culturing	Phenotypic identification	Potter et al., 1998
			CRC	19	11				
		Tissue samples	Control	23	4				
			Normal CRC	19	5.5				
			Tumor CRC	19	11				
		Blood	Control	23	0				
			CRC	19	0				
Malaysia	CRC patients with (bac+) and without bacteremia (bac−) and controls in tumor (TU) and non-tumor (NTU) specimens	Stool	Control	50	12	8% *SGG*	Selective culturing and DNA-based detection^*^	Phenotypic identification *sodA* gene PCR and qPCR	Abdulamir et al., 2010
			CRC bac−	52	13	10% *SGG*			
			CRC bac+	39	13	10% *SGG*			
		Mucosal washes	Control	50	6	4% *SGG*			
			CRC bac− (TU)	52	10	8% *SGG*			
			CRC bac− (NTU)	52	6	4% *SGG*			
			CRC bac+ (TU)	39	5	5% *SGG*			
			CRC bac+ (NTU)	39	5	5% *SGG*			
		Tissue specimens	Control	50	2	2% *SGG*			
			CRC bac− (TU)	52	17	17% (32.7%)^*^SGG			
			CRC bac− (NTU)	52	12	12% (23.0%)^*^SGG			
			CRC bac+ (TU)	39	21	21% (48.7%)^*^SGG			
			CRC bac+ (NTU)	39	13	13% (35.9%)^*^SGG			
Malaysia	Hospital population with CRC, inflammatory bowel disease and chronic gastrointestinal tract diseases (cases) and healthy individuals (controls)	Stool	Controls	96	7	n.d.	Selective culturing	Not described	Al-Jashamy et al., 2010
			IBD & chronic GID	29	52				
			CRC & adenoma	41	46				
France	Colonoscopy patients. Not selected for disease	Stool	Normal colonoscopy	134	6	0.4% *SGG*	Selective culturing	16S rRNA gene	Chirouze et al., 2013
			Non-tumoral lesions	76	1	0.7% *SGP*			
			Adenoma & carcinoma	49	6	3.5% *SL*			
Brazil	Colonoscopy patients in hospital	Rectal swab	Colonoscopy	54	35	11% *SGG*	Culture-independent	qPCR targeting *recN* and *gyrB*	Lopes et al., 2014
						13% *SGP*			
						20% *SL*			
						11% *SII*			
Israel	Colonoscopy patients. Acceptable indications for colonoscopy included screening for colorectal malignancy, postpolypectomy surveillance, and investigation of symptoms including hematochezia, abdominal pain, and change in bowel habits	Stool, aspirated colonic fluid and colonic tissue	Normal colonoscopy	105	12	n.d.	Selective culturing	VITEK2	Boltin et al., 2015
			Non-advanced or advanced lesions	13	15	n.d.			
South Africa	(Cohort 1) Patients with CRC without pre-selecting conditions	Tissue	Adenocarcinoma and adjacent normal	55	0	Not detected	Culture-independent	*sodA* gene qPCR	Viljoen et al., 2015
	(Cohort 2) Patients with sporadic microsatellite instability	Tissue	Adenocarcinoma	18	0	Not detected			
Spain	Unselected CRC patients	Matched tissue	Normal mucosa	190	0	Not detected	Culture-independent	*sodA* gene qPCR	Andres-Franch et al., 2017
			Tumor	190	6	3% *SGG*			
Germany	General population	Stool	Healthy controls	99	63	63% *SGG*	Culture-independent	qPCR targeting *recN*	Dumke et al., 2017
Kenya	Colonoscopy patients. Not selected for disease	Rectal swab	Normal colonoscopy	193	14	8% *SII*	Selective culturing	16S rRNA gene	Kaindi et al., 2017
			Adenomas & carcinomas	80	22	22% *SII*		*groEL* sequencing	
USA	Tumor and normal adjacent tissue	Tissue	Adjacent normal	128	47	47% *SGG*	Culture-independent	16S rRNA gene qPCR	Kumar et al., 2017
			Carcinoma	148	74	74% *SGG*			

*bac+, patients with bacteremia; bac−, patients without bacteremia; GID, gastrointestinal disorders; IBD, inflammatory bowel disease; n.d., not determined; NTU, non-tumor tissue specimen; TU, tumor tissue specimen*.

### Selective colonization of CRC patients and preliminary causal evidence

#### Selective colonization of CRC patients

Fecal carriage of SBSEC members associated with CRC was initially reported to be 56% in 63 CRC patients compared to 11% in 105 healthy controls (Table 1) (Klein et al., 1977). This association has since been reported in a range of 6–46% in patients with adenomas and CRC vs. 7–14% in control patients (Abdulamir et al., 2010; Al-Jashamy et al., 2010; Chirouze et al., 2013; Boltin et al., 2015; Kaindi et al., 2017). Of these five studies, only two observed a significant carriage difference between healthy and neoplasia patients but one suggested a novel association of *SII* prevalence with hemorrhoids (Al-Jashamy et al., 2010; Kaindi et al., 2017).

The selective association with CRC tissue is also controversial. While no specific association of SBSEC members with CRC tissue was observed on small sample sets based on phenotypic identification (Norfleet and Mitchell, 1993; Potter et al., 1998), more recent DNA-based approaches approve of such an association ranging from 0 to 2% in controls, 47% in normal tissue of cancer patients and 3–74% in tumor tissues (Table 1) (Abdulamir et al., 2010; Andres-Franch et al., 2017; Kumar et al., 2017). Interestingly, tumor tissue of patients with bacteremia was more often positive for *SGG* (48.7%) than that of patients without bacteremia (32.7%) based on *sodA* gene PCR, which increased the detection rate of *SGG* from 27% and 16%, respectively, based on selective culturing. (Abdulamir et al., 2010). Tumor tissues harboring *SGG* were also significantly associated with co-infection by Epstein-Barr virus (OR: 9.49; 95% CI: 1.1–82.9) (Andres-Franch et al., 2017). Nevertheless, two other studies using a culture-based and qPCR-based approach still suggested no association between tumorous (0–15%) and non-tumorous tissues (0–12%) for *SB*/*SGG* (Boltin et al., 2015; Viljoen et al., 2015).

These contradicting data on SBSEC tumor tissue colonization might relate to patients with clinical infections of SBSEC (Abdulamir et al., 2010) or without any clinical symptoms (Viljoen et al., 2015; Andres-Franch et al., 2017), differences in detection techniques (culture-independent vs. selective culturing) or study population. This also indicates a non-obligatory relationship of *SGG* or SBSEC with CRC. SBSEC might only proliferate if certain requirements to facilitate colonization are met to become passengers as hypothesized in the bacterial-driver-passenger model (Tjalsma et al., 2012).

#### Preliminary causality of SBSEC in CRC

Only minimal evidence for a causal relationship between SBSEC and CRC exists. Wall-extracted antigens from *SGG* NCTC8133, a human fecal isolate with controversial earlier classification as *SII* or *SE*, and its active S300 fraction are suggested to induce cell proliferation, polyamines and aberrant crypt foci in the distal colon of azoxymethane-treated rats (Ellmerich et al., 2000b; Biarc et al., 2004; Jans et al., 2016). In human CRC cell lines, stationary *SGG* strains TX20005 and TX20030 increased cell proliferation of HCT116, HT-29 and LoVo but not of SW480, SW1116, human lung carcinoma cell line A549, kidney epithelial cell line HEK293, human normal colon epithelial cell lines CCD841, CoN, and FHC suggesting the need for specific conditions to facilitate cell proliferation (Kumar et al., 2017).

The adenomatous polyposis coli tumorsuppressor gene *APC* is inactivated in many CRCs. This leads to an accumulation of β-catenin in the nucleus (He et al., 1998) thereby activating the c-Myc oncogene. Increased levels of β-catenin and c-Myc were detected in CRC cells after *SGG* incubation. This effect was not observed for *SII, SGP*, and *SGM* or for live *SGG* separated by trans-well membranes from cells, nor with bacterial supernatants, heat-killed bacteria or bacterial lysates suggesting a necessity of live *SGG* and direct *SGG*-cell contact (Kumar et al., 2017).

In azoxymethane-treated mice, inoculation with *SGG* led to increased cell proliferation, β-catenin accumulation in colonic crypts and higher numbers of tumors and dysplasia grade supporting a potential tumor-promoting role for *SGG* (Kumar et al., 2017). This effect was only observed for *SGG* strains able to induce cell proliferation *in vitro* (Kumar et al., 2018). The increase in cell proliferation was correlated with increased adhesion abilities *in vitro*. *In vivo* such strains also had an increased ability to colonize the mucosa in both C57BL6 as A/J mice. Therefore, the original bacterial driver-passenger model (Tjalsma et al., 2012) might need to be updated regarding the potential driving role of *SGG*. If *SGG* contributes to tumor development it would exert its effects probably after colonization of neoplastic sites as a passenger, which might depend on the specific strain and contribute to tumor progression rather than tumor initiation (Boleij and Tjalsma, 2013). In fact, the presence of polyps in the intestinal tract of Notch/*APC* mice allowed colonization and persistence of *SGG* UCN34 colonization, which was 1,000-fold higher than Notch control mice (Aymeric et al., 2018). However, no preferential adherence to tumor tissue sites was observed. Colonization was evenly distributed through the ileum and proximal colon, stimulated by secondary bile acids and was dependent on bacteriocins BlpA and BlpB that compete with other enterococci in the gut (Aymeric et al., 2018).

Interestingly, *in vitro* cultivation experiments suggested a growth advantage for *SGG* and *SGM* in the tumor microenvironment. Cultivation of *SGG* UCN34 and *SGM* CIP105683^T^ in spent medium from CRC cells HT-29, SW480, HCT116, and Caco-2 was used to simulate utilization of metabolites from the tumor microenvironment (Boleij et al., 2012a). *SGG* and *SGM* displayed significantly increased growth rates in spent medium of Caco-2 cells whereas the growth rates of e.g., *Salmonella enterica* subsp. *enterica* serovar Typhimurium, *Staphylococcus lugdunensis* or *Enterobacter cloacae* were reduced, suggesting a significant advantage for SBSEC to proliferate in the spent CRC metabolites. The major changes in protein expression patterns were related to an upregulation of pyrimidine biosynthesis and glycolysis, particularly glycerolypid, glycosis and fructose utilization; and a downregulation of purine metabolism. Furthermore, *SGG* seems to be specifically capable to use secondary glucose metabolites fructose 6-phosphate and 3-phosphate glyceric acid (Boleij et al., 2012a). As the tumor microenvironment features increased levels of lactate, glucose derivatives, amino acids, lipids and fatty acids, *SGG* likely has an advantage to proliferate in this niche (Boleij et al., 2012a). Therefore, *SGG* can be described as an opportunistic pathobiont benefiting from the favorable oncogenic environment to colonize the host. This eventually promotes its translocation and systemic dissemination, in select cases leading to clinical infections.

## Transmission and niche adaption of SBSEC members

### Transmission of SBSEC between hosts

The prevalence in the GIT of animals and humans facilitates transmission between animals and humans via feces and saliva (Dumke et al., 2015, 2017). Over a duration of 4 weeks, *SB* counts of an estimated 10^7^ CFU/g broiler feed and 10^8^ CFU/g wheat straw were only reduced by one log unit indicating high transmission possibility (Guy et al., 1980; Mackey and Hinton, 1990). Furthermore, soil clay adhesion of *SB* from bovine feces is very strong and cannot be desorbed after 24 h whereas long-term persistence seems weak but possibly sufficient to establish transmission within shorter time frames as observed among poultry flocks, surrounding environment and workers. In laying hens, colonization of non-carrier birds introduced into an *SGG*-positive flock took approximately 35 weeks and occurred likely via feed and feces (Guy et al., 1980; Dumke et al., 2015; Schulz et al., 2015). *SGG* isolates of identical sequence types where thereby causing infection in one worker and contributing to in-flock and old-young transmission in hens (Dumke et al., 2015). Similarly, sequence types were shared between turkey, pigeons, chicken and humans (Schulz et al., 2015). Rural residency, close animal-human contact and the use of manure as fertilizer were identified as risk factors for colonization with *SGG, SGP, SII, SL*, and *SB* in humans (Giannitsioti et al., 2007; Corredoira et al., 2008; Dumke et al., 2017).

Transmission via the fecal-oral or food-oral route requires survival of the gastric passage for colonization of the gut. Survival to simulated gastric conditions is dependent on the *SB* strain, gastric pH and feed ingestion. At pH < 2.5, survival is minimal whereas at pH 3.0 a reduction of approximately 3 log units was observed for *SII* while *SGM* did not survive, suggesting species-specific abilities to survive and reach the intestine in sufficient numbers for colonization (Ripamonti et al., 2011; Jans et al., 2016). The role of transmission via fermented food products that contain *SII, SL*, or *SGM* particularly in sub-Saharan Africa, Southern Europe, Asia and Latin America is not yet clear and niche adaptation might play a pivotal role in their ability to colonize the host (Jans et al., 2015, 2017; Kaindi et al., 2017).

### Niche adaptation of SBSEC members

SBSEC members adapt to multiple niches. In the rumen, the main carbon sources are largely available in the form of plant fibers. Their utilization is a key feature of many SBSEC members. Proteolysis and peptidase activity are important for the breakdown of proteins to ammonia (Wallace and McKain, 1991) that further supports growth of SBSEC members (Sales-Duval et al., 2002). α- galactosidase, β-glucanases, and endoglucanases of SBSEC play important roles for the degradation of complex carbohydrates in ruminants and chicken (Bailey, 1963; Ekinci et al., 1997; Beckmann et al., 2006). Most of these enzymes are adapted to GIT conditions with a narrow pH optimum of pH 5.6–6.3 and a temperature optimum of 37–42°C (Bailey, 1963; Wang et al., 2015; Chen et al., 2016), except for α-amylase and lactate dehydrogenase activity that increases at pH 5.5 vs. pH 6.5 and is possibly part of a self-feeding loop for lactate overproduction (Chen et al., 2016). At pH > 6.0, metabolism is directed toward production of formate, acetate and ethanol (Chen et al., 2016), while below pH 5.5, it is directed toward lactate, particularly in the event of excess glucose sources such as starch (Gunsalus and Niven, 1942; Russell and Hino, 1985; Asanuma and Hino, 1997). Ruminal acidosis results in a drop below pH 5.5 causing bloat in the rumen (Penner et al., 2007). Despite inconclusive causality, evidence suggests initiation by the overgrowth of *SB* in combination with *Prevotella, Ruminococcus, Streptococcus*, and *Lactobacillus* and the parallel inability of lactate utilizers such as *Megasphaera elsdenii* and *Selenomonas ruminantium* to metabolize lactate (Palmonari et al., 2010; Wang et al., 2015; McCann et al., 2016).

Particularly *SGG* has retained the ability to utilize a wide range of carbon sources typical for the rumen. This feature is minimized in *SGM* and *SII* or modified to a different variety of carbon sources in *SGP* (Rusniok et al., 2010; Lin et al., 2011b; Papadimitriou et al., 2014). *SGP* ATCC43144 in contrast to *SGG* ATCC43143 harbors a α-L-rhamnosidase, several endo-β-N-acetylglucosaminidase, glucokinase, glucosidases, mannosidases to utilize specific carbon sources available in the gut originating from plant cell walls, biofilms, glycosides, and glycolipids (Lin et al., 2011b). *SGG* is the only *Streptococcus* known so far to use malate via the malolactic enzyme (Gallo_2048) and a malate transporter (Gallo_2049) as well as degrade tannins encoded by *tanA*, that are otherwise toxic to many bacteria (Rusniok et al., 2010; Papadimitriou et al., 2014). *SGG* UCN34 is also able to hydrolyze bile salts, an important feature to survive in the small intestine. *SGG* is prototroph for all 20 amino acids. Besides, *SGG* harbors partial biosynthesis pathways for biotin and thiamine to support growth in varying conditions including the rumen, intestine and also the blood system (Rusniok et al., 2010).

Niche adaptation is also observed in *SII, SGM*, and *SGP* via gene loss and gain. They cannot biosynthesize pantothenate and biotin (Lin et al., 2011b; Papadimitriou et al., 2014) but depend on them for growth (Barnes et al., 1961). Dairy variants of *SII* strains adapted to the dairy environment via a modified lactose (LacS and LacZ instead of phosphotransferase) and peptide metabolism (duplication of oligopeptide transporters) (Jans et al., 2012a, 2013). Dairy *SGM* lost or harbor truncated genes for degradation of plant carbohydrates and detoxification of substances relevant for survival in the rumen that are likely obsolete in the dairy niche. In addition, dairy *SGM* gained gene clusters for casein hydrolysis, lactose and galactose metabolism for optimal utilization of these milk components (Papadimitriou et al., 2014). Both dairy *SII* and *SGM* possess lactocepin with high sequence similarity to PrtS CEP of *S. thermophilus* responsible for milk protein degradation. Furthermore, *SGP* showed gene loss likely related to adaptation to nutrient-rich environments and an overall genome reduction compared to *SGG* (Papadimitriou et al., 2014). These findings demonstrate the high adaptability of SBSEC members to different carbon and protein sources not only within the GIT, but also within the dairy environment.

Niche adaptability is also reflected by a high genome plasticity among SBSEC members. The pangenome is increasing at a high rate entailing numerous unique genes for each new strain added (Hinse et al., 2011b). Nearly all SBSEC genomes reveal horizontal gene transfer relating to general carbohydrate metabolism, capsular polysaccharides, antimicrobial resistance or tannase as the key discriminator of *SGG* (Rusniok et al., 2010; Hinse et al., 2011b; Lin et al., 2011b; Jans et al., 2013; Papadimitriou et al., 2014; Kambarev and Caté, 2015; Grimm et al., 2017a; Kambarev et al., 2017). Natural competence operons and pseudopilus identified in *SII* and *SGG* likely contribute to the success of horizontal gene transfer and thus to niche adaptation (Rusniok et al., 2010; Lin et al., 2011b; Morrison et al., 2013; Jans et al., 2016). As a consequence, SBSEC members feature highly diverse metabolic abilities and likely also different virulence factors depending on the species and different impact depending on the niche colonized.

## Mechanisms and virulence factors responsible for adhesion and host colonization by SBSEC members

The establishment of bacteria in a niche depends on a multitude of factors relating to adherence, signaling, nutritional adaptation and host modulation. Some of the key factors involved in adhesion and colonization in streptococci include cell-wall anchored factors such as LPXTG-motif proteins, anchorless factors including the cell capsule, two-component signal transduction systems for signaling or released/secreted factors such as exopolysaccharides to form biofilms (Nobbs et al., 2009; Brouwer et al., 2016).

Employing epithelial and endothelial cell lines provides an advanced model to study adhesion via bacteria-cell interactions. Adhesion capabilities can vary depending on the environment and the associated microbiota as well as the cell type present. Given the presence of SBSEC members in GIT and the blood system, adhesion and colonization have to be comprehensively assessed using designated cell lines. This includes cells originating from oral (primary buccal epithelial cells), gastric (rumen epithelial cell line), intestinal (human CRC cell lines Caco-2, HT-29, HCT116 and mucus-producing HT-29 MTX; mouse rectum carcinoma CMT-93) and venous sites (human vascular endothelial EA.hy926, human umbilical vein HUVEC, unnamed saphenous vein and mouse endothelial tumor EOMA).

### Adhesion to epithelial cell lines of the GIT

Oral epithelial cells represent the first cells in the GIT to interact with SBSEC members upon ingestion. Adhesion to buccal epithelial cells of human IE-derived *SB* biotype I and II strains was around 2–3 times higher than that of commensal reference strain *SB* DSM20480^T^ = NCTC8177 (Von Hunolstein et al., 1993), suggesting that epithelial adhesion is particularly present among IE-derived strains and likely dose-dependent (Ellmerich et al., 2000a).

Adhesion in the rumen seems to be pH and cell type dependent. Highest adhesion of *SB* strains to rumen epithelial cells was observed between pH 7.0–7.3. Near ruminal pH of 6.5, adhesion was still elevated suggesting that these *SB* strains adapted to the rumen (Styriak et al., 1994; Wang et al., 2015). An important factor for adhesion in the rumen was related to epithelial keratinization and particularly glycocalix (a glycoprotein and glycolipid cell surface layer) present on differentiated cells (Table 2). Keratinization significantly enhanced adherence of *SB* and enabled adhesion for those isolates unable to adhere to non-keratinized cells (Semjén and Gálfi, 1990; Styriak et al., 1991, 1992). However, host-specificity of *SB* strains using sheep and calve rumen epithelial cells was inconclusive. Therefore, it remains unclear whether host-specificity is a driving factor in rumen colonization (Semjén and Gálfi, 1990; Styriak et al., 1992).

**Table 2:** Adhesion of SBSEC species to cell lines.

	**SBSEC species with adhesion % (no. of strains adhesion positive/total no. of strains)**							
**Cell line**	***SGG***	***SGM***	***SGP***	***SL***	***SB***	**Isolation sources**	**Comments**	**References**
**EPITHELIAL CELL LINES**								
Bovine rumen epithelial cell line not differentiated	–	–	–	–	100% (2/2)	Bovine rumen	Keratinization positively influenced adhesion. Adhesion stronger to bovine than ovine cells	Semjén and Gálfi, 1990
Bovine rumen epithelial cell line non-keratinized	–	–	–	–	0% (0/4)	Bovine rumen		Styriak et al., 1992
	–	–	–	–	0% (0/3)	Bovine rumen	Biotype II	Styriak et al., 1994
Bovine rumen epithelial cell line keratinized	–	–	–	–	100% (4/4)	Bovine rumen		Styriak et al., 1992
	–	–	–	–	100% (3/3)	Bovine rumen	Biotype II	Styriak et al., 1994
Ovine rumen epithelial cell line	–	–	–	–	100% (2/2)	Bovine rumen	Keratinization positively influenced adhesion. Adhesion stronger to bovine than ovine cells.	Semjén and Gálfi, 1990
Ovine rumen epithelial cell line non-keratinized	–	–	–	–	20% (1/5)	Bovine rumen		Styriak et al., 1991
	–	–	–	–	0% (0/4)	Bovine rumen		Styriak et al., 1992
Ovine rumen epithelial cell line keratinized	–	–	–	–	100% (5/5)	Bovine rumen		Styriak et al., 1991
	–	–	–	–	100% (4/4)	Bovine rumen		Styriak et al., 1992
Oral/primary buccal epithelial cells	100% (3/3)	–	–	–	100% (2/2)	Human IE		Von Hunolstein et al., 1993
	–	–	–	–	100% (1/1)	Bovine feces	*SB* NCTC8177, adhesion significantly weaker than human IE strains	Von Hunolstein et al., 1993
**CRC CELL LINES**								
Human Caco-2	100% (2/2)	–	–	–	–	Bovine feces		Sánchez-Díaz et al., 2016
	100% (2/2)	–	–	–	–	Human blood		Sánchez-Díaz et al., 2016
	100% (1/1)	–	–	–	–	Human feces	*SGG* NCTC8133, dose-dependent binding	Ellmerich et al., 2000a
	–	–	100% (1/1)	–	–	Human blood	*SGP* ATCC43144, dose-dependent binding	Ellmerich et al., 2000a
	100% (3/3)	–	–	–	–	Human blood	*SGG* UCN34, 1293, NTB1	Boleij et al., 2011b
	100% (1/1)	–	–	–	–	Human feces	*SGG* NCTC8133	Boleij et al., 2011b
	–	100% (1/1)	–	–	–	Dairy	*SGM* CIP105865^T^	Boleij et al., 2011b
	–	–	–	18% (3/17)	–	Sea otter IE		Counihan et al., 2015
	–	–	–	0% (0/1)	–	Sea otter feces		Counihan et al., 2015
	–	–	–	0% (0/1)	–	Sea otter brain		Counihan et al., 2015
	–	–	–	0% (0/1)	–	Sea otter lymph		Counihan et al., 2015
	–	–	–	100% (1/1)	–	Sea otter ulcer		Counihan et al., 2015
Human epithelioid carcinoma cell line KB ECACC 86103004	100% (1/1)	–	–	–	–	Human feces	*SGG* NCTC8133, dose-dependent binding, discontinued cell line due to HeLa derivation	Ellmerich et al., 2000a
	–	–	100% (1/1)	–	–	Human blood	*SGP* ATCC43144, dose-dependent binding	Ellmerich et al., 2000a
Human HCT116	100% (2/2)	–	–	–	–	Human IE	*SGG* TX20005, TX20030	Kumar et al., 2017
Human HT-29	100% (3/3)	–	–	–	–	Human blood	*SGG* UCN34, 1293, NTB1	Boleij et al., 2011b
	100% (1/1)	–	–	–	–	Human feces	*SGG* NCTC8133	Boleij et al., 2011b
	–	100% (1/1)	–	–	–	Dairy	*SGM* CIP105865^T^	Boleij et al., 2011b
	100% (1/1)	–	–	–	–	Human IE	*SGG* UCN34	Martins et al., 2015
	100% (5/5)	–	–	–	–	Human IE	*SGG* TX20005, TX20030, TX20031 (promoting cell proliferation); *SGG* TX20008, TX20034, ATCC43143 (non-promoting cell proliferation). Adhesion lower for non-promoting strains	Kumar et al., 2018
	100% (2/2)	–	–	–	–	Human IE	*SGG* TX20005, TX20030	Kumar et al., 2017
Human HT-29 MTX	100% (1/1)	–	–	–	–	Human IE	higher adhesion to HT-29 MTX vs. HT-29	Martins et al., 2015
Mouse rectum carcinoma CMT-93	–	–	–	6% (1/17)	–	Sea otter IE		Counihan et al., 2015
	–	–	–	0% (0/1)	–	Sea otter feces		Counihan et al., 2015
	–	–	–	0% (0/1)	–	Sea otter brain		Counihan et al., 2015
	–	–	–	0% (0/1)	–	Sea otter lymph		Counihan et al., 2015
	–	–	–	0% (0/1)	–	Sea otter ulcer		Counihan et al., 2015
Human SW1116	100% (2/2)	–	–	–	–	Human IE	*SGG* TX20005, TX20030	Kumar et al., 2017
Human SW480	100% (2/2)	–	–	–	–	Human IE	*SGG* TX20005, TX20030	Kumar et al., 2017
**ENDOTHELIAL CELL LINES**								
Human 494 umbilical vein HUVEC	–	–	–	41% (7/17)	–	Sea otter IE		Counihan et al., 2015
	–	–	–	0% (0/1)	–	Sea otter feces		Counihan et al., 2015
	–	–	–	0% (0/1)	–	Sea otter brain		Counihan et al., 2015
	–	–	–	0% (0/1)	–	Sea otter lymph		Counihan et al., 2015
	–	–	–	0% (0/1)	–	Sea otter ulcer		Counihan et al., 2015
Mouse endothelial tumor EOMA	–	–	–	47% (8/17)	–	Sea otter IE		Counihan et al., 2015
	–	–	–	0% (0/1)	–	Sea otter feces		Counihan et al., 2015
	–	–	–	100% (1/1)	–	Sea otter brain		Counihan et al., 2015
	–	–	–	0% (0/1)	–	Sea otter lymph		Counihan et al., 2015
	–	–	–	100% (1/1)	–	Sea otter ulcer		Counihan et al., 2015
Human unnamed saphenous vein	100% (1/1)	–	–	–	–	Human feces	*SGG* NCTC8133, dose-dependent binding	Ellmerich et al., 2000a
	–	–	100% (1/1)	–	–	Human blood	*SGP* ATCC43144, dose-dependent binding	Ellmerich et al., 2000a
Human vascular endothelial EA.hy926	100% (21/21)	–	–	–	–	Human clinical IE/blood/other		Vollmer et al., 2010
	100% (1/1)	–	–	–	–	Koala feces	**SGG** DSM 16831^T^	Vollmer et al., 2010
	100% (1/1)	–	–	–	–	Shea cake digester	*SGG* DSM13808	Vollmer et al., 2010
**OTHER CELL LINES**								
Human lung carcinoma A549	100% (2/2)	–	–	–	–	Human IE	*SGG* TX20005, TX20030	Kumar et al., 2017
Human normal colon epithelial cell line CCD 841 CoN	100% (2/2)	–	–	–	–	Human IE	*SGG* TX20005, TX20030, adherence positive but significant lower than to cancer lines	Kumar et al., 2017
THP−1 human monocytes	100% (1/1)	–	–	–	–	Human feces	*SGG* NCTC8133, dose-dependent binding	Ellmerich et al., 2000a
	–	–	100% (1/1)	–	–	Human blood	*SGP* ATCC43144, dose-dependent binding	Ellmerich et al., 2000a

In the colon, adhesion of SBSEC members was assessed using epithelial CRC cell lines CMT-93, Caco-2 and HT-29 (Table 2). Adhesion to these CRC cell lines was observed for several SBSEC species including *SL, SGG* and *SGM*. Animal-derived *SL* strains (sea otter IE, feces, brain and lymph node isolates) adhered highly variable to CMT-93 and Caco-2. Significant adhesion (>0.2–0.3% of the inoculum) to CMT-93 and Caco-2 was rare within this strain pool and at slightly lower levels than the *S*. Typhimurium reference strain (Counihan et al., 2015). Adhesion to CMT-93 was generally lower than and without correlation to that of Caco-2 even for *S*. Typhimurium (Counihan et al., 2015) suggesting low suitability of CMT-93 cells for SBSEC adhesion assessment and possibly the need to evaluate sea otter-derived *SL* for host specificity.

Adhesion abilities toward Caco-2 and HT-29 of human-derived *SGG* and dairy *SGM* was low (<15% of inoculum) to intermediate (20–50% of inoculum) for *SGG* and *SGM/SE*, respectively (Table 2). Low adhesion was comparable with that of *S*. Typhimurium whereas intermediate adhesion was comparable to that of *E. coli* and *Lb. plantarum* reference strains, but significantly lower than the 80–98% observed for *E. faecalis*. Differences were particularly evident for *SGG* NCTC8133 that more efficiently adhered to Caco-2 than HT-29 (Boleij et al., 2011b). A comparative assessment of blood-derived *SGG* of IE patients indicated significantly enhanced adhesion abilities to HT-29 cells, particularly among strains able to promote tumor cell proliferation (Kumar et al., 2018). This differentiation between proliferation-promoting and non-promoting *SGG* was paralleled by the ability to colonize mice. Interestingly, mice colonization was increased in A/J type mice compared to C57BL/6 mice, which might be related to different host factors required for colonization (Kumar et al., 2018). Therefore, certain tumor-promoting strains might possess enhanced adhesion capabilities and thus a selective advantage particularly in a tumor environment presenting favorable factors. General adhesion of SBSEC members to CRC cell lines however seems limited in comparison to other gut pathogens.

### Adhesion to endothelial cell lines

Similar to epithelial cell lines, dose-dependent adhesion behavior was also observed for *SGG* NCTC8133 (human fecal isolate) and *SGP* ATCC43144 (human blood isolate) to human saphenous vein endothelial cell lines (Table 2). Especially at low inoculums, binding to endothelial cells was higher than to epithelial cells suggesting a preference toward endothelium (Ellmerich et al., 2000a). This was also observed among primarily human IE *SGG* isolates adhering to EA.hy926 cells but not with animal feces-derived *SGG* DSM16831^T^ (Vollmer et al., 2010). Mechanical stress on HUVEC cells had no influence on adherence suggesting that *SGG* actively colonizes endothelial tissues (Vollmer et al., 2010). Interestingly, only blood isolates of *SGG* express the blood-group antigen sialyl lewis-X (sLe^x^) on their cell surface. sLe^x^ is normally expressed on the cell surface of leukocytes enabling rolling of leukocytes on the endothelium. This might increase *SGG* adhesion to endothelial cells (Hirota et al., 1996).

Significant adhesion to endothelial HUVEC-C cells was observed in multiple IE and septicemia *SL* strains isolated from sea otters (Counihan et al., 2015). The ability to adhere to cell lines other than HUVEC-C was minimal; only one strain showed adhesion to human epithelial Caco-2, human endothelial HUVEC-C and mouse endothelial tumor EOMA cells (Table 2). The other strains showed minimal adhesion to mouse/human epithelial or endothelial cells, suggesting key differences in adhesion mechanisms to different cell types and possibly host origin. In general, isolates from heart or blood adhered better to intact endothelial cells and support possible IE establishment without previous history of heart disease (Counihan et al., 2015). However, general adhesion of *SL* strains was in the range of 0.05–0.2% of the inoculum and thus significantly lower than the 2% of human clinical *Staphylococcus aureus* ATCC25923 used as reference (Counihan et al., 2015). The biological impact to trigger IE despite this significantly lower adhesion ability of *SL* in contrast to *S. aureus* will require further evaluation in relation to host specificity and mechanisms responsible for endothelial tissue colonization by SBSEC members *in vitro* and *in vivo*.

### Binding to extracellular matrix proteins

Extracellular matrix proteins (ECM) are an important component to facilitate bacterial binding to epithelial and endothelial cell surfaces and thus niche colonization in humans and animals. Collagen thereby plays an important role. Collagen type I is present in organ capsules and scar tissue, such as on damaged heart valves. Collagen type IV is the main constituent of basement membranes and can become exposed at tumor sites (Tanjore and Kalluri, 2006; Boleij and Tjalsma, 2013).

Adhesion to collagen type I and IV is a key feature of *SGG* (Table 3). Most *SGG* strains derived from human blood cultures of IE or bacteremia patients, pigeons suffering from streptococcosis (*SGG* and *SGP*) and *SL* strains derived from sea otters with IE, but also fecal and dairy SBSEC isolates displayed binding to collagen type IV. In contrast, binding to collagen type I was a feature mostly associated with human blood-derived *SGG, SGP, SII* and *SL* isolates and partially also with *SGM* isolates (Vanrobaeys et al., 2000a; Sillanpää et al., 2008; Vollmer et al., 2010; Boleij et al., 2011b; Counihan et al., 2015; Grimm et al., 2018). Among a panel of human and food-derived *SII* and *SL* as well as dairy *SGM*, adhesion to collagen type I and IV was particularly present in human blood isolates (Boleij et al., 2011b; Jans et al., 2016). In contrast, *SGG* considered as commensals and isolated from human feces, pigeons and ruminants including the *SGG* type strain rarely bound to collagen type I, III and IV (Table 3). Similar differences were also observed between *SGG* from infected vs. *SGG* from healthy humans featuring high vs. low adhesion, respectively (Grimm et al., 2018). *SGG* NCTC8133 and *SGP* strain ATCC43144 were only shown to bind collagen type IV whereas *SGP* DSM15351^T^ strains displayed no adhesion to collagen type I and IV. Other collagen types bound by human IE-derived *SGG* are collagen type II (96%) and to a lesser extent collagen type V (40%) (Table 3) (Ellmerich et al., 2000a; Sillanpää et al., 2008; Vollmer et al., 2010). Differences in collagen adhesion patterns therefore seem to exist between animal and human-derived strains even within the same species. Whether these different adhesion patterns among *SGG* and other SBSEC members also translate into different abilities to cause disease remains to be investigated.

**Table 3:** Adhesion of SBSEC species to different extracellular matrix protein (ECM) types.

	**SBSEC species with adhesion % (no. of strains adhesion positive/total no. of strains)**							
**ECM (source)**	***SGG***	***SGM***	***SGP***	***SII***	***SL***	***SB***	**Isolation source**	**References**
**COLLAGEN**								
Type I (calf skin)	0% (0/9)	–	0% (0/5)	–	–	–	Pigeon streptococcosis	Vanrobaeys et al., 2000a
Type I (human)	25% (6/24)	–	–	–	–	–	animal	Grimm et al., 2018
	0% (0/1)	–	–	–	–	–	Food	Grimm et al., 2018
	78% (35/45)	–	–	–	–	–	Human blood	Grimm et al., 2018
	0% (0/2)	–	–	–	–	–	Human feces	Grimm et al., 2018
	0% (0/2)	–	–	–	–	–	Unknown	Grimm et al., 2018
Type I (rat tail)	100% (1/1)	–	–	–	0% (0/1)	–	Animal	Jans et al., 2016
	100% (2/2)	–	–	–	–	–	Bovine feces	Sánchez-Díaz et al., 2016
	–	60% (3/5)	–	10% (3/29)	–	–	Dairy	Jans et al., 2016
	–	–	–	0% (0/1)	–	–	Food contamination	Jans et al., 2016
	–	–	–	75% (3/4)	100% (1/1)	–	Human blood	Jans et al., 2016
	100% (2/2)	–	–	–	–	–	Human blood	Sánchez-Díaz et al., 2016
	–	–	0% (0/1)	–	–	–	Human cerebrospinal fluid	Jans et al., 2016
	100% (1/1)	–	–	–	0% (0/4)	–	Human feces	Jans et al., 2016
	73% (11/15)	–	100% (1/1)	–	0% (0/1)	–	Human IE	Sillanpää et al., 2008
	–	–	–	0% (0/5)	–	–	Human unknown	Jans et al., 2016
Type I (source n/a)	100% (23/23)	–	–	–	–	–	Human IE and others^*^	Vollmer et al., 2010
	100% (3/3)	–	–	–	–	–	Human IE	Boleij et al., 2011b
	100% (1/1)	–	–	–	–	–	Animal feces	Boleij et al., 2011b
	–	100% (1/1)	–	–	–	–	Dairy	Boleij et al., 2011b
Type II (source n/a)	96% (22/23)	–	–	–	–	–	Human IE and others^*^	Vollmer et al., 2010
Type III (calf skin)	22% (2/9)	–	0% (0/5)	–	–	–	Pigeon streptococcosis	Vanrobaeys et al., 2000a
Type IV (human)	100% (1/1)	–	–	–	0% (0/1)	–	animal	Jans et al., 2016
	100% (2/2)	–	–	–	–	–	Bovine feces	Sánchez-Díaz et al., 2016
	–	20% (1/5)	–	14% (4/29)	–	–	Dairy	Jans et al., 2016
	100% (1/1)	–	–	–	–	–	Food	Grimm et al., 2018
	–	–	–	0% (0/1)	–	–	Food contamination	Jans et al., 2016
	–	–	–	75% (3/4)	100% (1/1)	–	Human blood	Jans et al., 2016
	–	–	0% (0/1)	–	–	–	Human cerebrospinal fluid	Jans et al., 2016
	0% (0/1)	–	–	–	0% (0/4)	–	Human feces	Jans et al., 2016
	60% (9/15)	–	0% (0/1)	–	0% (0/1)	–	Human IE	Sillanpää et al., 2008
	–	–	–	0% (0/5)	–	–	Human unknown	Jans et al., 2016
	38% (9/24)	–	–	–	–	–	Animal	Grimm et al., 2018
	82% (37/45)	–	–	–	–	–	Human blood	Grimm et al., 2018
	100% (2/2)	–	–	–	–	–	Human blood	Sánchez-Díaz et al., 2016
	0% (0/2)	–	–	–	–	–	Human feces	Grimm et al., 2018
	0% (0/2)	–	–	–	–	–	Unknown	Grimm et al., 2018
Type IV (mouse sarcoma)	89% (8/9)	–	100% (5/5)	–	–	–	Pigeon streptococcosis	Vanrobaeys et al., 2000a
Type IV (mouse tumor)	–	–	–	–	100% (10/10)	–	Sea otter brain^§^, feces^§^, IE, ulcer^§^	Counihan et al., 2015
Type IV (source n/a)	–	–	100% (1/1)	–	–	–	Human blood	Ellmerich et al., 2000a
	100% (1/1)	–	–	–	–	–	Human feces	Ellmerich et al., 2000a
	96% (22/23)	–	–	–	–	–	Human IE and others^*^	Vollmer et al., 2010
	100% (3/3)	–	–	–	–	–	Human IE	Boleij et al., 2011b
	100% (1/1)	–	–	–	–	–	Animal feces	Boleij et al., 2011b
	–	100% (1/1)	–	–	–	–	Dairy	Boleij et al., 2011b
Type V (human)	40% (6/15)	–	0% (0/1)	–	0% (0/1)	–	Human IE	Sillanpää et al., 2008
**OTHER ECM SOURCES**								
Fibrinogen (human)	100% (1/1)	–	–	–	0% (0/1)	–	Animal	Jans et al., 2016
	–	40% (2/5)	–	7% (2/29)	–	–	Dairy	Jans et al., 2016
	–	–	–	0% (0/1)	–	–	Food contamination	Jans et al., 2016
	–	–	–	75% (3/4)	100% (1/1)	–	Human blood	Jans et al., 2016
	–	–	0% (0/1)	–	–	–	Human cerebrospinal fluid	Jans et al., 2016
	100% (1/1)	–	–	–	0% (0/4)	–	Human feces	Jans et al., 2016
	47% (7/15)	–	100% (1/1)	–	0% (0/1)	–	Human IE	Sillanpää et al., 2008
	78% (18/23)	–	–	–	–	–	Human IE and others^*^	Vollmer et al., 2010
	–	–	–	0% (0/5)	–	–	Human unknown	Jans et al., 2016
Fibronectin (human)	100% (1/1)	–	–	–	0% (0/1)	–	Animal	Jans et al., 2016
	–	–	–	–	–	20% (2/10)	Bovine rumen	Styriak et al., 1999
	–	60% (3/5)	–	7% (2/29)	–	–	Dairy	Jans et al., 2016
	–	–	–	0% (0/1)	–	–	Food contamination	Jans et al., 2016
	–	–	–	75% (3/4)	100% (1/1)	–	Human blood	Jans et al., 2016
	–	–	0% (0/1)	–	–	–	Human cerebrospinal fluid	Jans et al., 2016
	0% (0/1)	–	–	–	0% (0/4)	–	Human feces	Jans et al., 2016
	33% (5/15)	–	100% (1/1)	–	0% (0/1)	–	Human IE	Sillanpää et al., 2008
	–	–	–	0% (0/5)	–	–	Human unknown	Jans et al., 2016
	67% (6/9)	–	60% (3/5)	–	–	–	Pigeon streptococcosis	Vanrobaeys et al., 2000a
	–	–	–	–	100% (10/10)	–	Sea otter brain^§^, feces^§^, IE, ulcer^§^	Counihan et al., 2015
Fibronectin (porcine)	–	–	–	–	–	0% (0/10)	Bovine rumen	Styriak et al., 1999
Fibronectin (source n/a)	–	–	100% (1/1)	–	–	–	Human blood	Ellmerich et al., 2000a
	100% (1/1)	–	–	–	–	–	Human feces	Ellmerich et al., 2000a
	30% (7/23)	–	–	–	–	–	Human IE and others^*^	Vollmer et al., 2010
Heparin	–	–	–	–	–	0% (0/10)	Bovine rumen	Styriak et al., 1999
Lactoferrin (bovine)	–	–	–	–	–	20% (2/10)	Bovine rumen	Styriak et al., 1999
Laminin (mouse tumor)	–	–	–	–	100% (10/10)	–	Sea otter brain^§^, feces^§^, IE, ulcer^§^	Counihan et al., 2015
Laminin (source n/a)	–	–	100% (1/1)	–	–	–	Human blood	Ellmerich et al., 2000a
	100% (1/1)	–	–	–	–	–	Human feces	Ellmerich et al., 2000a
	70% (16/23)	–	–	–	–	–	Human IE and others^*^	Vollmer et al., 2010
Mucin type II (porcine stomach)	100% (1/1)	–	–	–	100% (1/1)	–	Animal	Jans et al., 2016
	–	40% (2/5)	–	17% (5/29)	–	–	Dairy	Jans et al., 2016
	–	–	–	0% (0/1)	–	–	Food contamination	Jans et al., 2016
	–	–	–	100% (4/4)	100% (1/1)	–	Human blood	Jans et al., 2016
	–	–	0% (0/1)	–	–	–	Human cerebrospinal fluid	Jans et al., 2016
	0% (0/1)	–	–	–	0% (0/4)	–	Human feces	Jans et al., 2016
	–	–	–	0% (0/5)	–	–	Human unknown	Jans et al., 2016
Tenascin	70% (16/23)	–	–	–	–	–	Human IE and others^*^	Vollmer et al., 2010
Vitronectin (human)	–	–	–	–	–	20% (2/10)	Bovine rumen	Styriak et al., 1999
Vitronectin (source n/a)	22% (5/23)	–	–	–	–	–	Human IE and others^*^	Vollmer et al., 2010
Bovine serum albumin (BSA)	0% (0/1)	–	–	–	0% (0/1)	–	Animal	Jans et al., 2016
	–	–	–	–	–	0% (0/10)	Bovine rumen	Styriak et al., 1999
	–	40% (2/5)	–	10% (3/29)	–	–	Dairy	Jans et al., 2016
	–	–	–	0% (0/1)	–	–	Food contamination	Jans et al., 2016
	–	–	–	75% (3/4)	100% (1/1)	–	Human blood	Jans et al., 2016
	–	–	0% (0/1)	–	–	–	Human cerebrospinal fluid	Jans et al., 2016
	0% (0/1)	–	–	–	0% (0/4)	–	Human feces	Jans et al., 2016
	0% (0/15)	–	0% (0/1)	–	0% (0/1)	–	Human IE	Sillanpää et al., 2008
	–	–	–	0% (0/5)	–	–	Human unknown	Jans et al., 2016
	–	–	–	–	100% (10/10)	–	Sea otter brain^§^, feces^§^, IE, ulcer^§^	Counihan et al., 2015
Human serum albumin (HSA)	–	–	–	–	–	0% (0/10)	Bovine rumen	Styriak et al., 1999

* *23 strains total of which 2 originated from fecal samples of a koala bear (type strain) and one anaerobic shea cake digester isolate, but data was not extractable by strain*.

§ *data derived from single strains*.

Connective tissue and the tumor-microenvironment contain an extensive network of ECM including collagen, laminins, fibronectin, proteoglycans, and hyaluronans (Peddareddigari et al., 2010). *SL* strains from sea otters adhered to fibronectin, laminin, and hyaluronic acid in all cases (Table 3) (Counihan et al., 2015). Also human blood-derived *SII* and *SL* featured high adhesion abilities to fibronectin (Jans et al., 2016). Interestingly, the *SL* genome features relevant hits to adhesion factors such as pneumococcal cell surface adherence protein A PavA involved in fibronectin-binding and the laminin-binding protein Lmb (Jin et al., 2013). Binding to fibronectin is also observed in the *SGG* type strain and the majority of pigeon-derived *SGG* strains (Table 3). Interestingly, fibronectin-binding is less prevalent in human-derived *SGG* and *SGP* with the exception of *SGG* NCTC8133 and human blood strain *SGP* ATCC43144 (Ellmerich et al., 2000a; Vanrobaeys et al., 2000a; Sillanpää et al., 2008; Vollmer et al., 2010; Jans et al., 2016). Furthermore, rumen-derived *SB* strains showed low or no binding to both human and porcine fibronectin (Styriak et al., 1999). Similar, the *SII* type strain, *SII* dairy and *SL* human commensal strains showed with a few exceptions only minor adhesion abilities to fibronectin, collagen type I and IV, mucin and fibrinogen (Table 3) (Jans et al., 2016).

Fibrinogen-binding is in contrast to fibronectin-binding a common feature also among human IE *SGG* strains, dairy *SGM*, human *SII* and *SL* blood isolates (Table 3) (Ellmerich et al., 2000a; Sillanpää et al., 2008; Vollmer et al., 2010; Jans et al., 2016). Human IE-derived *SGG* also showed interactions with tenascin, laminin and vitronectin (Ellmerich et al., 2000a; Sillanpää et al., 2008; Vollmer et al., 2010). In contrast, rumen *SB* strains showed mostly moderate or weak adhesion to bovine lactoferrin, vitronectin, heparin, and BSA. None of the *SB* strains bound to human serum albumin (Table 3) (Styriak et al., 1999). These differences in ECM adhesion patterns between animal and human strains might therefore be important for their colonization abilities of different body sites. These patterns furthermore suggest different adhesion mechanisms in *SL, SGG* and other SBSEC members (Lin et al., 2011b; Papadimitriou et al., 2014). Particularly human and animal blood isolates seem to have the ability to bind fibrinogen, while fibronectin-binding is variable, which implies different adhesion abilities regarding fibronectin in the tumor-microenvironment and fibrinogen at damaged sites requiring blood clotting.

### Biofilm formation, exopolysaccharides, dextran production and capsular polysaccharides

Adhesion and biofilm-forming abilities are linked to colonization and persistence in the GIT. *SB* produce at least two types of polysaccharides: (1) water-soluble glucans, often dextrans, comprised of α-1:6 linked glucose units (Bailey, 1959); and (2) capsular polysaccharide (Bailey and Oxford, 1958). GtfA in *SGG* was found to produce water-insoluble α-1,3-linked glucosidic polymers whereas GtfB encoded for α-1,3-linked water-insoluble and α-1,6-linked glucosidic water-soluble polymers (Lin et al., 2011b). Both types of polysaccharides have specific roles in adhesion, colonization and host immune evasion (Nobbs et al., 2009; Isenring et al., 2018). The ability to form biofilms is however not directly correlated to virulence and needs to be carefully distinguished (Vollmer et al., 2010).

Biofilm formation was observed with SBSEC strains from GIT, blood and food origin (Vollmer et al., 2010; Boleij et al., 2011b; Jans et al., 2016). *SII* and other SBSEC members were also observed to form biofilms on human teeth featuring various degrees of auto- and co-aggregation with other oral microbes (Shen et al., 2005; Arul and Palanivelu, 2014). Even outside a host, biofilm formation to uncoated plastic and stainless steel surfaces was observed for all SBSEC species of blood, animal and dairy origin (Flint et al., 1997, 1999; Jans et al., 2016).

Polysaccharides are major constituents of biofilms (Christensen, 1989; Nobbs et al., 2009). *SGG, SL* and many SBSEC members are known to produce extracellular glucan encoded by glycosyl-transferases similar to GtfA, GtfB, and GtfC of *S. mutans* but lacking in *SGP* ATCC43144 (Rusniok et al., 2010; Lin et al., 2011b). Instead, *SGP* harbored a strain-specific exopolysaccharide biosynthesis gene cluster featuring sequence identity highest with those of *Bacillus cereus* and *Clostridium thermocellum* (Lin et al., 2011b). Generally, this suggests that from a common SBSEC ancestor, *SGG* likely kept most biofilm-related loci while the respective loci were either absent or comprised of pseudogenes in *SGP, SII* and *SGM* potentially reducing or abrogating biofilm formation capabilities in comparison to *SGG* (De Vuyst and Tsakalidou, 2008; Lin et al., 2011b; Papadimitriou et al., 2014). Biofilm production might therefore be SBSEC-species dependent but older data are inconclusive in this respect.

Dextran production from rumen *SB* biotype II isolates of sheep, calve and cow is particularly dependent on available sugar compounds and a CO_2_ source. In contrast to capsular polysaccharides, the production of dextran is limited and directly correlated with the available sucrose concentration (Bailey and Oxford, 1958; Barnes et al., 1961; Cheng et al., 1976). The CO_2_ source can include ${\text{HCO}}_{3}^{-}$, which is readily available in the rumen (Bailey and Oxford, 1958; Barnes et al., 1961). For dextran production by *SB*, three different growth requirements are suggested: (i) biotin and ammonium chloride as sole vitamin and N-source, respectively, (ii) calcium, pantothenate, adenine, biotin, thiamine and arginine or glutamic acid, or (iii) xanthine and additional amino acids (Barnes et al., 1961). Prototroph *SGG* in contrast to *SB* biotype II likely possess the metabolic capabilities to produce biofilm even in niches not meeting these growth requirements (Rusniok et al., 2010).

Dextran production furthermore seems to play a role in ruminal acidosis (Humer et al., 2018). It is hypothesized that the higher sucrose content of grain feed boosts dextran production in ruminants to form a slime in the rumen (Cheng et al., 1976; Kulp and Ponte, 2000; Humer et al., 2018). This slime, comprised of proteins and polysaccharides of other bacteria, increases viscosity and produces a froth foam eventually leading to bloat (Cheng et al., 1976). A key role in the slime production process is attributed to *SB* via acidification and dextran production via its rumen-adapted dextran sucrase (Bailey and Oxford, 1958; Min et al., 2006).

### Virulence factors of SBSEC members related to adhesion and colonization

#### General aspects of the cell surface in relation to adhesion

The bacterial cell surface has important roles in the interaction with the environment, the host and for pathogenesis (Nobbs et al., 2009; Isenring et al., 2018). Lipoproteins featuring a serine-rich motif following a cysteine residue are frequently present on the surface of *SGG* UCN34, possibly linked to specific interactions with polysaccharides from the environment (Rusniok et al., 2010). Wall-extracted antigens from the cell surface of SBSEC members bound equally well to epithelial and endothelial cell lines as whole SBSEC cells, supporting a role for cell surface factors in adhesion (Ellmerich et al., 2000a). Among surface proteins, *SB* surface protein Sbs6, Sbs10, Sbs13, and Sbs16 as well as the histone-like protein HlpA, autolysin AtlA and the cell surface protein antigen C PaC are currently characterized SBSEC virulence factors besides pili. While HlpA is present in most SBSEC members, Sbs13, Sbs16, AtlA and PaC are mainly limited to *SGG* whereas Sbs6 and Sbs10 are also regularly observed in *SE*. Among *SGG*, only blood-derived *SGG* usually feature all seven surface proteins in contrast to rumen or fecal isolates (Table 4 and Supplementary Data 1). This suggests reduced or different virulence characteristics of the other SBSEC members in comparison to blood-derived *SGG*.

**Table 4:** Presence of major known virulence factors of SBSEC members in relation to adhesion, colonization and immune system interaction for SBSEC strains of human, animal and food origin.

**Species**	**Category**	**No of strains**	***pil1*** **locus**			***pil2*** **locus**			***pil3*** **locus**			***sbs6***	***sbs10***	***sbs13***	***sbs16***	***hlpA***	***atlA***	***PAc***
			**Sortase C-type**	**Fimbrial protein (LPXTG motif) Pil1B**	**Collagen-binding protein Pil1A**	**Sortase C-type**	**Fimbrial protein (LPXTG motif) Pil2B**	**Putative Adhesin TQXA domain-containing protein**	**Sortase C-type**	**Cell wall anchor (LPXTG motif) Pil3B**	**Peptidoglycan linked protein (LPXTG motif) Pil3A**	**Cell envelope proteinase A**	**Glycosyl hydrolase family 32**	**TQXA domain-containing protein**	**Collagen-binding protein**	**Surface exposed histone-like protein A**	**Autolysin**	**Cell surface protein antigen C**
*SE*	Animal feces	3	+ (1); − (2)	+ (1); − (2)	+ (0); − (2); other (1)	+ (3); − (0)	+ (3); − (0)	+ (0); − (1); truncated (1); other (1)	+ (3); − (0)	+ (3); − (0)	+ (3); − (0)	+ (2); − (1)	+ (3); − (0)	+ (0); − (3)	+ (0); − (3)	+ (3); − (0)	+ (0); − (0); other (3)	+ (0); − (3)
*SE*	Animal rumen	9	+ (3); − (6)	+ (3); − (6)	+ (0); − (6); other (3)	+ (7); − (2)	+ (7); − (2)	+ (0); − (6); other (3)	+ (7); − (2)	+ (7); − (2)	+ (7); − (2)	+ (6); − (3)	+ (5); − (4)	+ (0); − (9)	+ (0); − (9)	+ (6); − (3)	+ (0); − (4); other (5)	+ (0); − (9)
*SE*	Animal unknown	8	+ (8); − (0)	+ (8); − (0)	+ (0); − (0); other (8)	+ (7); − (1)	+ (7); − (1)	+ (0); − (6); other (2)	+ (8); − (0)	+ (8); − (0)	+ (8); − (0)	+ (5); − (3)	+ (5); − (3)	+ (2); − (6)	+ (0); − (8)	+ (8); − (0)	+ (2); − (1); other (5)	+ (2); − (6)
*SE*	Human feces	2	+ (0); − (2)	+ (0); − (2)	+ (0); − (2)	+ (1); − (1)	+ (1); − (1)	+ (0); − (1); truncated (1)	+ (1); − (1)	+ (0); − (1); truncated (1)	+ (1); − (1)	+ (1); − (1)	+ (1); − (1)	+ (0); − (2)	+ (0); − (2)	+ (2); − (0)	+ (1); − (0); other (1)	+ (0); − (2)
*SE*	Human other	1	+ (0); − (1)	+ (0); − (1)	+ (0); − (1)	+ (0); − (1)	+ (0); − (1)	+ (0); − (1)	+ (0); − (1)	+ (0); − (1)	+ (0); − (1)	+ (1); − (0)	+ (1); − (0)	+ (1); − (0)	+ (1); − (0)	+ (1); − (0)	+ (1); − (0)	+ (0); − (1)
*SE*	Unknown	8	+ (4); − (4)	+ (4); − (4)	+ (0); − (6); other (2)	+ (6); − (2)	+ (6); − (2)	+ (0); − (5); other (3)	+ (8); − (0)	+ (8); − (0)	+ (8); − (0)	+ (7); − (1)	+ (6); − (2)	+ (0); − (8)	+ (0); − (8)	+ (7); − (1)	+ (3); − (1); other (4)	+ (0); − (8)
*SGG*	Animal feces	2	+ (0); − (2)	+ (1); − (1)	+ (0); − (1); truncated (1)	+ (1); − (1)	+ (1); − (1)	+ (1); − (1)	+ (2); − (0)	+ (2); − (0)	+ (2); − (0)	+ (1); − (1)	+ (1); − (1)	+ (2); − (0)	+ (2); − (0)	+ (2); − (0)	+ (2); − (0)	+ (1); − (1)
*SGG*	Animal other	2	+ (2); − (0)	+ (2); − (0)	+ (0); − (0); other (2)	+ (1); − (0); truncated (1)	+ (2); − (0)	+ (0); − (0); truncated (1); other (1)	+ (2); − (0)	+ (2); − (0)	+ (2); − (0)	+ (0); − (2)	+ (0); − (2)	+ (0); − (0); other (2)	+ (2); − (0)	+ (2); − (0)	+ (1); − (1)	+ (0); − (2)
*SGG*	Animal rumen	6	+ (3); − (3)	+ (6); − (0)	+ (0); − (0); other (6)	+ (2); − (4)	+ (5); − (1)	+ (2); − (4)	+ (4); − (2)	+ (4); − (2)	+ (4); − (2)	+ (6); − (0)	+ (3); − (3)	+ (4); − (2)	+ (2); − (4)	+ (6); − (0)	+ (6); − (0)	+ (6); − (0)
*SGG*	Human blood	6	+ (6); − (0)	+ (6); − (0)	+ (6); − (0)	+ (6); − (0)	+ (6); − (0)	+ (6); − (0)	+ (6); − (0)	+ (6); − (0)	+ (6); − (0)	+ (6); − (0)	+ (6); − (0)	+ (6); − (0)	+ (6); − (0)	+ (6); − (0)	+ (6); − (0)	+ (4); − (2)
*SGG*	Human feces	2	+ (0); − (2)	+ (0); − (2)	+ (0); − (2)	+ (0); − (2)	+ (0); − (2)	+ (0); − (2)	+ (1); − (1)	+ (1); − (1)	+ (1); − (1)	+ (0); − (2)	+ (0); − (2)	+ (0); − (2)	+ (0); − (2)	+ (2); − (0)	+ (2); − (0)	+ (0); − (2)
*SGM*	Food	2	+ (0); − (2)	+ (0); − (2)	+ (0); − (2)	+ (0); − (2)	+ (0); − (2)	+ (0); − (2)	+ (1); − (1)	+ (1); − (1)	+ (1); − (1)	+ (0); − (2)	+ (0); − (2)	+ (0); − (2)	+ (0); − (2)	+ (2); − (0)	+ (1); − (0); other (1)	+ (0); − (2)
*SGP*	Human blood	1	+ (0); − (1)	+ (0); − (1)	+ (0); − (1)	+ (0); − (1)	+ (0); − (1)	+ (0); − (1)	+ (1); − (0)	+ (1); − (0)	+ (1); − (0)	+ (0); − (1)	+ (0); − (1)	+ (1); − (0)	+ (0); − (1)	+ (1); − (0)	+ (1); − (0)	+ (0); − (1)
*SGP*	Unknown	3	+ (0); − (3)	+ (0); − (3)	+ (0); − (3)	+ (0); − (3)	+ (0); − (3)	+ (0); − (3)	+ (0); − (3)	+ (0); − (3)	+ (0); − (3)	+ (0); − (3)	+ (0); − (3)	+ (0); − (3)	+ (0); − (3)	+ (1); − (2)	+ (1); − (2)	+ (0); − (3)
*SII*	Food	1	+ (0); − (1)	+ (1); − (0)	+ (0); − (1)	+ (0); − (1)	+ (0); − (1)	+ (0); − (1)	+ (1); − (0)	+ (1); − (0)	+ (1); − (0)	+ (1); − (0)	+ (0); − (1)	+ (0); − (1)	+ (0); − (1)	+ (1); − (0)	+ (0); − (0); other (1)	+ (0); − (1)
*SII*	Human feces	2	+ (0); − (2)	+ (1); − (1)	+ (0); − (2)	+ (0); − (2)	+ (0); − (2)	+ (0); − (2)	+ (1); − (1)	+ (1); − (1)	+ (1); − (1)	+ (1); − (1)	+ (0); − (2)	+ (0); − (2)	+ (0); − (2)	+ (1); − (1)	+ (1); − (1)	+ (0); − (2)
*SL*	Animal other	1	+ (0); − (1)	+ (0); − (1)	+ (0); − (1)	+ (0); − (1)	+ (0); − (1)	+ (0); − (1)	+ (0); − (1)	+ (0); − (1)	+ (0); − (1)	+ (0); − (1)	+ (0); − (1)	+ (1); − (0)	+ (0); − (1)	+ (1); − (0)	+ (1); − (0)	+ (0); − (1)
*SL*	Human feces	1	+ (0); − (1)	+ (0); − (1)	+ (0); − (1)	+ (0); − (1)	+ (0); − (1)	+ (0); − (1)	+ (0); − (1)	+ (0); − (1)	+ (0); − (1)	+ (0); − (1)	+ (0); − (1)	+ (0); − (1)	+ (0); − (1)	+ (1); − (0)	+ (0); − (0); other (1)	+ (0); − (1)
Locus Tag			Gallo_2177	Gallo _2178	Gallo _2179	Gallo _1568	Gallo _1569	Gallo _1570	Gallo _2038	Gallo _2039	Gallo _2040	Gallo _0748	Gallo _0112	Gallo _2032	Gallo _0577	Gallo _0636	Gallo _1368	Gallo _1675
Reference protein sequence size (AA)			275	480	658	275	505	641	312	478	1664	1573	1301	775	750	91	992	759
Min. AA sequence match for ≪+≫			200	400	600	200	400	600	200	400	1000	1000	1000	500	500	50	500	500
Identity threshold (%)			50	50	50	50	50	50	50	50	50	50	50	50	50	50	50	50

*Data derived via a comparative blast search in 60 SBSEC genomes for the presence of virulence genes encoding for pili and adhesion-related proteins based on SGG UCN34 (strain-specific data available in Supplementary Data 1)*.

*Data derived from blast search using the protein sequences of the nine genes of pil1-3 and further virulence factors of SGG UCN34. A positive hit is indicated by “+” if the AA sequence is above the minimum sequence length determined for each locus and >50% AA sequence identity. “other” indicates AA sequences matching the minimum AA sequence length but <50% AA sequence identity. Genes with high identity but incomplete length are indicated as “truncated.” The numbers in brackets indicate the number of strains for each category in that specific row. Other terminology used for the pilus operons: pil1 as identified by Danne et al. (2011) refers to the acb-sbs7-srtC1 operon and pil3 refers to sbs15-sbs14-srtC2. acb stands for collagen binding adhesion bovis and sbs stands for S. bovis surface protein (Sillanpää et al., 2009)*.

Specific studies were performed on enolase and HlpA. Enolase is a conserved anchorless surface protein involved in cross-linking of *SGG* UCN34 and human epithelial cells (Boleij et al., 2011a). The main interaction partner was identified as cytokeratin 8. Cytokeratin 8 is constantly expressed by epithelial cells, but at increased levels by CRC and could therefore play a role in the association of *SGG* with CRC (Boleij et al., 2011a).

HlpA is highly prevalent among SBSEC members possibly involved in adhesion (Table 4) (Boleij et al., 2009a; Lin et al., 2011b; Papadimitriou et al., 2014). HlpA is an anchorless bacterial surface protein that binds to lipoteichoic acid at the Gram-positive cell wall. Lipoteichoic acid was previously suggested to be involved in adhesion in cooperation with surface proteins (Von Hunolstein et al., 1993; Styriak et al., 1994). Binding to colon tumor cells is then further established via heparan sulfate proteoglycans (Boleij et al., 2009a). However, heparin (a heparan sulfate proteoglycan) and lipoteichoic acid compete for the same binding sites in HlpA which cannot efficiently bind simultaneously to both structures (Boleij et al., 2009a) supporting earlier observations that heparin treatment of rumen *SB* isolates inhibited lactoferrin-binding (Styriak et al., 1999). ARH-77^syn^ myeloma cells overexpressing syndecan-1 (the predominant heparan sulfate proteoglycan on epithelial cells) displayed increased adherence of *SB*, other streptococci and staphylococci in contrast to ARH77 cells without syndecan-1 as well as *E. faecalis* or *E. coli* and other Gram-negative bacteria (Henry-Stanley et al., 2005). Therefore, heparan sulfate proteoglycans might play a significant role in epithelial interactions for staphylococci and streptococci to modulate interactions with tumor epithelial cells (Henry-Stanley et al., 2005; Boleij et al., 2009a).

#### The capsule

The capsular polysaccharide of SBSEC members primarily consists of galactose, rhamnose and uronic acid. It is produced from glucose or other carbohydrates and in contrast to exopolysaccharides does not need CO_2_ for production (Bailey and Oxford, 1958). Capsule properties are however strain dependent. Highly virulent strains, in this case only *SGG* strains, possessed a significantly thicker capsule whereas truncated genes in dairy isolate of *SII* or *SGM* might inhibit capsule production (Vanrobaeys et al., 1999; Boleij et al., 2011b; Papadimitriou et al., 2014). Genome data suggests a high diversity of capsular polysaccharides in *SGG, SGP* and *SII* varying in length between 12 and 19 genes with a conserved start followed by a strain-specific genetic variability. It is thus related to capsule heterogeneity and varying antigenic properties (Rusniok et al., 2010; Hinse et al., 2011b; Lin et al., 2011b; Jans et al., 2013; Jin et al., 2013; Papadimitriou et al., 2014).

The capsule of many streptococci also comprises hyaluronic acid, which is important for adhesion to host cells, colonization and phagocytic killing and infection (Hynes, 2004). Capsule degradation by hyaluronidase was significantly correlated with decreasing adherence to epithelial and endothelial cells (Counihan et al., 2015). Conversely, *SL* is also able to use host-derived hyaluronic acid to boost adherence and invasion suggesting a key role for hyaluronic acid in SBSEC pathogenesis (Counihan et al., 2015).

#### Pili and their role in adhesion

A key surface structure involved in the interaction of SBSEC members with their environment and hosts are fimbriae or pili. Initially, these specific surface structures were described as fimbriae among highly virulent *SGG* of pigeon origin (Vanrobaeys et al., 1999). However, it seems that currently recognized pili in SBSEC encompass these fimbriae. Fimbriae were therefore interpreted accordingly for this review.

In general, *SGG* harbors three pilus loci termed *pil1, pil2*, and *pil3*, each of which is comprised of three genes (Danne et al., 2011). The main exceptions among *SGG* seem to be animal-derived *SGG* including the type strain and human fecal *SGG*, which harbor incomplete pilus loci or significant mutations in the genes encoding for collagen-binding and adhesion in *pil1* and *pil2* (Table 4). In other SBSEC members, the three pilus loci display signs of genome decay through various mutated, truncated or completely absent loci and genes (Table 4 and Supplementary Data 1). Most *SE, SII* and *SGM* harbor only *pil3* as a complete locus with significant sequence identity to *SGG*. *pil1* and *pil2* loci are frequently incomplete due to truncated or completely absent genes. Similar to animal-derived *SGG*, the genes encoding for collagen-binding and adhesion in *pil1* and *pil2* are regularly mutated in *SE* and *SII*, which leaves open questions regarding their functional properties. *SL* and *SGP* do not seem to possess any structures resembling the pilus loci of *SGG* (Table 4 and Supplementary Data 1) (Jans et al., 2013; Papadimitriou et al., 2014). This might explain the absence of pili in low virulent *SGP* (Vanrobaeys et al., 1999) and suggests species-specific pilus organization. However, genome data is limited to animal commensal strains but not animal pathogens such as *SL* from sea otters or *SGP* from pigeons, which will be required to determine the pili and virulence factor repertoire in relation to SBSEC species and host.

The *pil1* locus in *SGG* is among the most investigated virulence factors of *SGG*. The three genes of *pil1* encode for two LPXTG-motif proteins and one sortase C. The two LPXTG-motif proteins [Gallo_2179 (Pil1A) and Gallo_2178 (Pil1B)] represent the typical features of pilin subunits with a pilin motif PK centralized in the protein containing a structural CnaB domain. Only Pil1A harbors a putative collagen-binding domain (Danne et al., 2011). The core pilus itself is comprised of heteropolymers of the two LPXTG-motif proteins while other parts were only comprised of Pil1A or Pil1B (Danne et al., 2011). A comparative blast search using the *pil1-3* locus proteins of *SGG* UCN34 to 60 available SBSEC genomes revealed that the collagen-binding protein Pil1A is unique among human blood *SGG* (Table 4 and Supplementary Data 1). This suggests that Pil1A is a key feature of *SGG* capable of causing IE that might have been lost or modified in other *SGG* lineages and SBSEC members. This highlights further that only some virulence factors specific for *SGG* have been unraveled whereas those specific for other SBSEC members remain mostly unknown.

Each pilus loci and the individual proteins seem to have specific roles of which only that of *pil1* and *pil3* have been elucidated. The role of the *pil1* pilus was confirmed to be a collagen-binding adhesin, important for biofilm formation and virulence, particularly in IE rat models using *SGG* UCN34 as model strain (Sillanpää et al., 2009; Danne et al., 2011). Adhesion assays confirmed preferred collagen I adhesion of *pil1* over collagen IV, fibronectin and fibrinogen. This finding is relevant for the establishment of biofilms by *SGG* on collagen-rich surfaces, which are observed in CRC tissues and on damaged heart valves where it may lead to IE development (Danne et al., 2011). *pil3* is a key factor in binding to mucus, which covers the intestinal epithelium (Lichtenberger, 1995). *In vivo* assays using *SGG* UCN34 displayed impaired colonization of Δ*pil3* mutants in the distal colon of mice (Martins et al., 2015). *pil3* also binds to human stomach mucins and human fibrinogen. This supports the importance of *pil3* in host adhesion by *SGG* UCN34 (Martins et al., 2016) and likely other *SGG, SII* and *SL* harboring *pil3* (Table 4), the presence of which correlated directly with fibrinogen and mucin adhesion *in vitro* (Jans et al., 2016; Isenring et al., 2018). This would give *pil1* and *pil3*-carrying *SGG* an advantage for the adhesion to CRC tissue featuring mislocalized MUC5AC mucin and exposed collagen type IV, the adhesion to fibrinogen at injured sites in the blood system as well as the colonization of collagen type I exposing surfaces such as damaged heart valves (Martins et al., 2016). This would subsequently initiate the first stage of IE.

## Invasion and infection establishment

SBSEC as pathobionts are opportunistic invasive pathogens able to colonize secondary sites via the bloodstream. SBSEC possess different mechanisms to establish disease by bacterial translocation, survival in the blood stream and adhesion to endothelial cell surfaces. In the case of underlying conditions such as damaged heart valves or CRC, SBSEC possess a selective advantage and cause severe bacteremia or IE in humans and animals.

### Epithelial cell response to host-microbe interaction

The intestinal epithelial barrier is an important line of defense of the GIT, as it is proposed to be the main point of entry to cause infection. Several studies have looked at the ability of SBSEC to be recognized by the epithelium and result in a local immune activation and chemoattraction. For example, *SGG* NCTC8133 and *SGP* ATCC 43144 have been shown to induce IL-8 secretion in buccal epithelial and endothelial cells in a dose-dependent manner, but not in intestinal Caco-2 cells. While *SGG* was more effective in the induction of IL-8 from buccal epithelial cells, *SGP* released higher levels of IL-8 from endothelial cells. Furthermore, *SE* and *SGM* induce IL-8 also at mRNA level, while *SGG* was not able to induce an IL-8 response in HT-29 and Caco-2 cells. None of the strains induced IL1-β at mRNA level in intestinal epithelial colon cells (Boleij et al., 2011b).

In contrast, wall-extracted antigens from *SE* are very effective in inducing IL-8 from buccal epithelial cells, endothelial cells and intestinal epithelial cells (Caco-2). Heat inactivation ablated the effect of wall-extracted antigens on IL-8 secretion, suggesting that proteinaceous components of the cell wall are involved in the IL-8 response (Ellmerich et al., 2000a). Especially the S300 fraction of the wall-extracted antigens was capable of inducing IL-8 and prostaglandin secretion in Caco-2 cells. Proteins in the wall-extracted antigens that could be responsible for these effects were GroEL, SOD, Dpr, Aldolase, Enolase and L-lactate dehydrogenase (Biarc et al., 2004). In contrast, colonization of pre-sensitized Balb/C mice with the *SB* HC5 strain producing the lantibiotic bovicin HC5, resulted in an increase influx of eosinofils and reduction of brushborder and goblet cells in the small intestine, with a 4-fold increase in cell proliferation, while the large intestine was not affected by this HC5 strain (Paiva et al., 2012).

### Epithelial/endothelial translocation and invasion

For bacterial invasion, either epithelial invasion or translocation is necessary to enter the human body. While adherence to epithelial cells ranged from 2 to 10% for *SGG*, 2 to 50% for *SE*, and 25 to 30% for *SGM* depending on the cell line used, internalization was below 0.1% for all strains (Boleij et al., 2011b; Sánchez-Díaz et al., 2016). *SGG* strains rarely showed significant invasion into epithelial cells (Table 5) (Sánchez-Díaz et al., 2016). The adhesion of *SL* to Caco-2 cells varied between 0.05 and 0.3%, and only one of 21 *SL* strains had a high invasion of 0.2% while all others were below 0.1% invasion in Caco-2 cells (Table 5) (Counihan et al., 2015). However, *SGG*, but not *SGM* and *SE*, was able to translocate across differentiated epithelial monolayers (Boleij et al., 2011b), suggesting species and strain-dependent abilities to invade and translocate epithelial cells as well as invade endothelial cells (Vollmer et al., 2010). Similarly, several *SL* isolates were able to invade HUVEC endothelial cells. In general, encapsulated strains were found to be more invasive, which further correlated with the presence of a hyaluronic acid capsule (Counihan et al., 2015). Some *SL* strains even have a mucoid appearance, which in other streptococci is associated with greater degrees of tissue necrosis and bacteremia (Counihan et al., 2015).

**Table 5:** Invasion and translocation ability of SBSEC species in different cell lines.

	**SBSEC species with invasion or translocation % (no. of strains invasion or translocation positive/total no. of strains)**					
**Cell line**	***SGG***	***SGM***	***SL***	**Isolation sources**	**Comments**	**References**
**Invasion**						
**CRC CELL LINES**						
Human Caco-2	0% (0/3)	–	–	Human blood	*SGG* UCN34, 1293, NTB1	Boleij et al., 2011b
	0% (0/1)	–	–	Human feces	*SGG* NCTC8133	Boleij et al., 2011b
	–	0% (0/1)	–	Dairy	*SGM* CIP105865^T^	Boleij et al., 2011b
	–	–	82% (14/17)	Sea otter IE	Only 1 strain with 0.2% invasion, all others low invasion	Counihan et al., 2015
	–	–	100% (1/1)	Sea otter feces	Low invasion	Counihan et al., 2015
	–	–	100% (1/1)	Sea otter brain	Low invasion	Counihan et al., 2015
	–	–	100% (1/1)	Sea otter lymph	Low invasion	Counihan et al., 2015
	–	–	100% (1/1)	Sea otter ulcer	Low invasion	Counihan et al., 2015
	0% (0/2)	–	–	Bovine feces	Low invasion	Sánchez-Díaz et al., 2016
	0% (0/2)	–	–	Human blood	Low invasion	Sánchez-Díaz et al., 2016
Human HCT116	0% (0/2)	–	–	Human IE	*SGG* TX20005, TX20030, considered as low to non-invasive	Kumar et al., 2017
Human HT-29	0% (0/2)	–	–	Human IE	*SGG* TX20005, TX20030, considered as low to non-invasive	Kumar et al., 2017
Human SW1116	0% (0/2)	–	–	Human IE	*SGG* TX20005, TX20030, considered as low to non-invasive	Kumar et al., 2017
Human SW480	0% (0/2)	–	–	Human IE	*SGG* TX20005, TX20030, considered as low to non-invasive	Kumar et al., 2017
**ENDOTHELIAL CELL LINES**						
Human 494 umbilical vein HUVEC	–	–	76% (13/17)	Sea otter IE	Low invasion	Counihan et al., 2015
	–	–	100% (1/1)	Sea otter feces	Low invasion	Counihan et al., 2015
	–	–	0% (0/1)	Sea otter brain	Low invasion	Counihan et al., 2015
	–	–	100% (1/1)	Sea otter lymph	Low invasion	Counihan et al., 2015
	–	–	100% (1/1)	Sea otter ulcer	Low invasion	Counihan et al., 2015
	100% (5/5)	–	–	Human clinical IE/blood/other		Vollmer et al., 2010
	0% (0/1)	–	–	Koala feces	*SGG* DSM16831^T^	Vollmer et al., 2010
Human vascular endothelial EA.hy926	100% (21/21)	–	–	Human clinical IE/blood/other		Vollmer et al., 2010
	0% (0/1)	–	–	Koala feces	*SGG* DSM16831^T^	Vollmer et al., 2010
	100% (1/1)	–	–	Shea cake digester	*SGG* DSM13808	Vollmer et al., 2010
**OTHER CELL LINES**						
Human lung carcinoma A549	0% (0/2)	–	–	Human IE	*SGG* TX20005, TX20030, considered as low to non-invasive	Kumar et al., 2017
Human normal colon epithelial cell line CCD 841 CoN	0% (0/2)	–	–	Human IE	*SGG* TX20005, TX20030, considered as low to non-invasive	Kumar et al., 2017
**Translocation**						
**CRC CELL LINE**						
Human Caco-2 epithelial monolayer	50% (1/2)	–	–	Bovine feces		Sánchez-Díaz et al., 2016
	100% (2/2)	–	–	Human blood		Sánchez-Díaz et al., 2016
	100% (3/3)	–	–	Human blood	*SGG* UCN34, 1293, NTB1	Boleij et al., 2011b
	100% (1/1)	–	–	Human feces	*SGG* NCTC8133	Boleij et al., 2011b
	–	0% (0/1)	–	Dairy	*SGM* CIP105865^T^	Boleij et al., 2011b

### Survival in blood and macrophages and activation of the human contact system

Survival in blood, activation of the human contact system and escape from the immune system are pivotal for infection establishment. For survival in blood, hemolysis can be a beneficial ability for bacteria to gain access to iron (Malachowa and DeLeo, 2011). Genome analysis of SBSEC members suggests the presence of multiple virulence factors related to hemolysis including hemolytic toxin CylZ as well as hemolysins TLY, III and A family protein (Jin et al., 2013; Papadimitriou et al., 2014). *In vitro* experiments on survival in blood however featured controversial data likely linked to SBSEC-species dependency. While serum isolated from heparin-treated blood had little bactericidal effect on most SBSEC strains, filament-forming *SB* strains had a much higher sensitivity to serum (Lorian and Atkinson, 1978). In contrast to heparin-treated blood, *SGG* and some *SII* demonstrate high survival and growth in citrate-treated blood similar to *Streptococcus pyogenes* AP1. Pil1 and Pil3 mutants seemed to have little effect on survival in blood whereas a capsule deficient mutant (Δ*cpsD*) showed reduced but not significant lower survival (Isenring et al., 2018).

*SGG*, but not *SII*, is able to activate the human contact system at the bacterial surface. The human contact system is comprised of a cascade of factors including serine proteases factor XI, XII and plasma prekallikrein. The co-factor high molecular weight kininogen and later plasma kallikrein are involved in degrading kininogen to liberate pro-inflammatory bradykinin (Isenring et al., 2018). This ability is linked to the presence of the *pil1* locus in the bacteria and the bacteria cell capsule encoded by multiple genes, one of which being *cpsD*. Pil1, particularly the Pil1A protein of this pilus, is able to bind factor XII and alter the host blood coagulation cascade. Mutants deprived of *pil1* or *pil3* showed decreased activity on factor XII and plasma kallikrein. An inverse correlation was observed for *cpsD*. *SGG* capsule mutant Δ*cpsD* showed increased activity on factor XII, plasma kallikrein and activated partial thromboplastin-time prolongation suggesting enhanced interaction between Pil1 and these factors if not hindered by the capsule. The interference with the human contact system and coagulation cascade by *SGG* suggests that Pil1 is an important factor for the establishment of IE in humans (Isenring et al., 2018).

Intracellular survival in macrophages and multiplication are important additional virulence mechanisms of multiple pathogens. *SGG* was able to survive in macrophages for up to 24 h and *SL* was shown to survive up to 48 h in macrophages (Counihan et al., 2015) whereas most *Lactobacillus, Lactococcus* and *Bacillus subtilis* are efficiently killed within this time-frame (Boleij et al., 2011b; Counihan et al., 2015). The presence of *pil1* and *cpsD* seems to affect macrophage survival. Non-piliated *SGG* mutants had high survival rates to phagocytosis whereas *pil1* overexpressing mutants and the Δ*cpsD* mutant were killed more efficiently (Isenring et al., 2018). This supports the protective effect of the capsule against phagocytosis and the impact of pilus expression and degree of piliation on immune system interactions (Danne et al., 2014; Martins et al., 2016). The addition of purified Pil1 antibodies increased the uptake of *pil1* overexpressing but not of Δ*pil1* bacteria by THP-1 macrophages. The opsonized *SGG* were mainly the *pil1* high expressing bacteria and not the weakly piliated cells. This suggests phase variation of *pil1* an important immune evasion mechanism to reach the blood system (Danne et al., 2014; Martins et al., 2015, 2016). Phase variation of *pil1* has important practical implications regarding silent infections. Patients can be asymptomatically affected by SBSEC bacteremia including the potential risk for undetected underlying CRC or adenomas (Haimowitz et al., 2005; Lin et al., 2011a; Lee et al., 2013). Pili are however not the only factor involved in immune system interaction. The rapid killing and cytokine induction of a non-piliated low virulent *SGG* strain lacking *pil1* and *pil3* (DSM16831^T^ isolated from koala feces) in contrast to the survival of *SGG* UCN34Δ*pil1* suggests that further factors besides *pil1* are involved in immune system interactions and the establishment of infection (Grimm et al., 2017b).

Pigeon and duckling infection models allow correlating the *in vitro* and *in vivo* findings. In the pigeon infection models, the highly virulent *SGG* strain STR357 was found intracellularly in 2–20% of macrophages counted in the spleen 96 h post-infection. *SGG* seemed to be actively multiplying. However, the exact mechanism of *SGG* to avoid killing by macrophages is not yet clear. Observations of free *SGG* outside phagosomes suggest a phagosome escape mechanism rather than the inhibition of phagosome-lysosome fusion (De Herdt et al., 1995).

In a duckling infection model, *SGP* resulted in meningitis and neurological symptoms 3 days post-infection. Macrophages were hypertrophic with abundant replicating *SGP* within phagosomes of degenerating macrophages causing necroptosis (Li et al., 2013). Based on these data *SGG, SGP*, and *SL* are capable of surviving in the blood stream, resisting phagocytosis and evading the immune system with strong dependency on the extracellular capsule, pili phase variation and so far unknown virulence factors.

### Causation of infective endocarditis

Native-valve IE is caused by the colonization of damaged endothelial tissue of heart valves by bacteria such as *Staphylococcus aureus*, enterococci or SBSEC members (Hoen and Duval, 2013). SBSEC members are suspected to cause significant endocardial endothelial damage (Schoemaker et al., 1994). The minimal infectious dose to reach a lethal dose (LD90) in an experimental rat IE model was determined to be 10^4^ CFU and is comparable to the LD90 of other IE pathogens (Danne et al., 2011). Important factors for the establishment of IE are binding of the bacteria to the endocardium and formation of a biofilm to shield microbes from immune attacks. The injured heart valve is recruited by platelets and fibrin, but also collagen was shown to be present in sterile injured valves. These sterile plaques (nonbacterial thrombotic endocarditis) can be colonized by pathogens during bacteremia. *SB* have a high adherence capability to normal valves, but an even 5-times increased binding to damaged aortic valves. Pil1 and Pil3 likely mediate this binding. Pil1, a collagen-binding adhesin, was shown to be important for IE establishment in a rat IE model and required for the initial establishment of an endocardial vegetation (Danne et al., 2011). Pil3 is of importance due to its ability to bind to fibrinogen. Due to platelet recruitment to injured sites, *pil3*-expressing strains have significantly improved ability to colonize the injured endocardium (Martins et al., 2016). Glucan-producing abilities of strains might further enhance binding to the endocardium. Treatment with dextranase inhibited the binding of glucan-positive strains, but not of glucan-negative strains, showing that the capsular glucan presence can be of significant importance for the binding efficiency of *SB* to injured sites (Ramirez-Ronda, 1978).

Further propagation of IE is linked again to the human contact system involving the coagulation cascade and the potent pro-inflammatory peptide bradykinin (Isenring et al., 2018). *SGG* UCN34 seems to prolong the intrinsic coagulation time by binding and activating factors of the human contact system on its surface. These findings were strongly related to Pil1. *SII* usually only possesses Pil3 explaining its inability to bind factor XII or plasma kallikrein, possibly reducing the overall impact of *SII* vs. *SGG* on coagulation time prolongation in the blood system. Likely, only wildtype *SGG* possess the capability to fully degrade kininogen in contrast to mutants with deletion of pili (Δ*pil1* or Δ*pil3*) or capsule (Δ*cpsD*) and overexpression of pil1 (Δ*term*). Since kininogen degradation leads to the release of bradykinin, also bradykinin release was significantly reduced in all mutant strains although not completely inhibited (Isenring et al., 2018). This supports the major role of Pil1 in the pathogenesis of *SGG*. Pathogenesis is likely also influenced by other factors including Pil3, glucan producing abilities and the capsule. Additional factors and their roles in pathogenesis in *SGG* and other SBSEC members, particularly that of Pil2, are still unknown. Based on current knowledge, this resulted in the postulation of the following IE model for *SGG*: *SGG* upon entering into the bloodstream can survive and multiply. Subsequently, the coagulation cascade is activated leading to a procoagulant state. The bacteria can then adhere to exposed collagen, most likely type I, on heart valves via Pil1-binding. This further activates contact factors at the bacterial surface and via the contact system cascade, leads to a release of bradykinin that binds to its receptor to trigger IE (Isenring et al., 2018).

## Clinical infections and host-immune response due to SBSEC in animals and humans

### Causation of IE and bacteremia

SBSEC members are placed among the top-five causes for human IE globally and responsible for up to 6% of confirmed IE cases (Hoen et al., 2005; Vogkou et al., 2016). SBSEC as causal agents of IE increased from 10% to over 20% of all streptococcal IE cases in Europe and the USA with a particular hotspot in France (Hoen et al., 2005). Of the SBSEC bacteremia cases, 47% developed into IE (Barnham and Weightman, 2004). SBSEC IE is particularly common in patients with congenital heart defects, prosthetic valves, heart-care associated cross infections, diabetes, living in rural areas and also increasingly affects healthy valves of subjects >65 years (Barrau et al., 2004; Corredoira et al., 2008; Vogkou et al., 2016).

*SGG* seems to be the main causative agent of IE in humans in comparison with *SGP, SII* and *SL*. Between seven to over 50% of cases are attributed to *SGG* followed by 17–30% to *SGP* and roughly 30% to *SL* with strong regional differences (Beck et al., 2008; Boleij et al., 2011c; Romero et al., 2011; Lazarovitch et al., 2013; Sheng et al., 2014; Marmolin et al., 2016; Ben-Chetrit et al., 2017).

SBSEC members are also of high importance for bacteremia and IE in animals (Jans et al., 2015). In pigeons, *SGG* and *SGP* were found as the etiological agent for bacteremia and IE causing spontaneous infections that result in liver-, kidney- and spleen-swelling (Devriese et al., 1990, 1998). The preferred route of infection is suggested to be the GIT (Kimpe et al., 2003). *SL* has been associated with bacteremia and IE in sea otters (Counihan-Edgar et al., 2012; Counihan et al., 2015) and is often isolated from the GIT, cardiac valve lesions, heart, blood, brain, and other organs of sea otters that died due to IE, but hardly ever from the GIT of healthy sea otters. Still, the GIT is suggested as the preferred route of entry for *SL* (Counihan et al., 2015). The disease shows high similarity with IE in humans, which suggests similar patterns in infection establishment, and thus the need for common concepts that help to identify SBSEC-specific virulence factors.

### Association of IE with underlying CRC and hepatobiliary disorders

SBSEC bacteremia and IE have a much stronger association with colon neoplasia than tumor colonization of SBSEC. A meta-analysis clearly indicated that especially *SGG* IE has a very high association with neoplasia (OR 7.26, CI 3.94–13.36) compared to *SB* biotype II species. The median prevalence of neoplasia in *SB* infected patients was 60%, and was higher for adenomas (43%) than for carcinomas (18%). *SB* infection could therefore be predominantly associated with premalignant colonic lesions, which underscores the importance of colon examination for colon pathologies (Boleij et al., 2009b, 2011c). This is supported by the fact that most neoplasias are identified during the episode of bacteremia in surveillance studies. Surveillance of *SGG* bacteremia patients resulted in 57 new neoplasias (seven carcinomas and 23 advanced adenomas) in 232 patients (24.6%) with a mean follow-up of 41.8 months. In *Clostridium septicum* bacteremia patients, no new neoplasias were found with a mean follow-up of 37.4 months (Corredoira et al., 2017). Retrospective analysis of patients with a previous *SB* bacteremia revealed the detection of three CRCs and seven adenomas in 14 patient that underwent colonoscopy (72% detection rate) (McKenna et al., 2011) strongly supporting the association of SBSEC IE with gut pathologies. This might support the theory that *SGG* bacteremia frequently has an intestinal source and is a marker for intestinal *SGG* colonization.

Stratified by SBSEC species (Table 6), *SGG* associates with colon pathology in 28.6–70.7% of cases varying from 16.1 to 52.5% for adenomas and 3.6–33.3% for carcinomas (Tripodi et al., 2004; Beck et al., 2008; Romero et al., 2011; Corredoira et al., 2013b, 2017; Lazarovitch et al., 2013; Sheng et al., 2014; Ben-Chetrit et al., 2017), *SGP* associates with 0.0–45.8% colon pathologies in 200 cases of which 126 were reported from Asia (Beck et al., 2008; Romero et al., 2011; Lazarovitch et al., 2013; Sheng et al., 2014; Ben-Chetrit et al., 2017). For *SL*, the data is more controversial ranging from 0.0 to 50.0% for colon pathologies, 21.4 and 33.3% reported by 2 studies for adenomas and only 1 study reported a rate of 7.1% for carcinomas. Similarly, data for *SII* and *SGM* are very limited showing an overall neoplasia rate of 14.3% for *SII* and 100% for *SGM*, often relying on a few single cases (Table 6) (Beck et al., 2008; Romero et al., 2011; Lazarovitch et al., 2013; Sheng et al., 2014; Ben-Chetrit et al., 2017). Meta-analysis of these studies together revealed colon pathology for SBSEC in 40.7% (*n* = 786) and divided by subspecies for *SGG* in 51.7% (*n* = 462), *SGP* in 24.5% (*n* = 200) and *SL* in 24.2% (*n* = 33) (Ben-Chetrit et al., 2017). However, in all these studies there was no control group reflecting the prevalence of colorectal adenomas in the general population >65 years of age that is estimated to be between 10 and 25% (Boleij et al., 2011c).

**Table 6:** Association of SBSEC subspecies clinical infections (bacteremia and IE) with underlying colon pathology.

		**Tripodi^†^**		Beck^*^		Romero^*^		**McKenna^‡^**		Lazarovitch^*^		**Corredoiran**		Sheng^*^		**Corredoira**		Ben-Chetrit^*^		**Total**	
		**2004**		**2008**		**2011**		**2011**		**2013**		**2013b**		**2014**		**2017**		**2017**			
**Identification**	**technique**	**phenotypic**		**16s rRNA**		**MALDI-TOF, 16s rRNA**, ***sodA***		**n.d**.		**VITEK 2**, ***groES/groEL***		**phenotypic, VITEK2**		**VITEK 2, 16s rRNA**, ***sodA, groES/groEL***		**API20 Strep, VITEK 2, 16s rRNA**, ***sodA***		**MALDI-TOF**			
		***n***	**%**	***n***	**%**	***n***	**%**	***n***	**%**	***n***	**%**	***n***	**%**	***n***	**%**	***n***	**%**	***n***	**%**	***n***	**%**
*SGG* cases		28		21		14				6		99		31		257		6		**462**	
	adenomas	13	46.4	5	23.8	3	21.4	–	–	–	–	52	52.5	5	16.1	103	40.1	1	16.7	182	39.4
	carcinomas	1	3.6	2	9.5	1	7.1	–	–	–	–	18	18.2	5	16.1	25	9.7	2	33.3	54	11.7
	neoplasia	14	50.0	7	33.3	4	28.6	–	–	3	50.0	70	70.7	10	32.3	128	49.8	3	50.0	239	51.7
*SGP* cases				11		24				13				126				26		**200**	
	adenomas	–	–	0	0.0	11	45.8	–	–	–	–	–	–	8	6.3	–	–	3	11.5	22	11.0
	carcinomas	–	–	0	0.0	0	0.0	–	–	–	–	–	–	19	15.1	–	–	4	15.4	23	11.5
	neoplasia	–	–	0	0.0	11	45.8	–	–	4	30.8	–	–	27	21.4	–	–	7	26.9	49	24.5
*SL* cases				14		5				4				4				6		**33**	
	adenomas	–	–	3	21.4	0	0.0	–	–	–	–	–	–	0	0.0	–	–	2	33.3	5	15.2
	carcinomas	–	–	1	7.1	0	0.0	–	–	–	–	–	–	0	0.0	–	–	0	0.0	1	3.0
	neoplasia	–	–	4	28.6	0	0.0	–	–	2	50.0	–	–	0	0.0	–	–	2	33.3	8	24.2
*SII* cases						2				1				11						**14**	
	adenomas	–	–	–	–	1	50.0	–	–	–	–	–	–	0	0.0	–	–	–	–	1	7.1
	carcinomas	–	–	–	–	0	0.0	–	–	–	–	–	–	0	0.0	–	–	–	–	0	0.0
	neoplasia	–	–	–	–	1	50.0	–	–	1	100.0	–	–	0	0.0	–	–	–	–	2	14.3
*SGM* cases																		2		**2**	
	adenomas	–	–	–	–	–	–	–	–	–	–	–	–	–	–	–	–	2	100.0	2	100.0
	carcinomas	–	–	–	–	–	–	–	–	–	–	–	–	–	–	–	–	0	0.0	0	0.0
	neoplasia	–	–	–	–	–	–	–	–	–	–	–	–	–	–	–	–	2	100.0	2	100.0
All SBSEC																					
cases		**30**		**46**		**45**		**37**		**24**		**135**		**172**		**257**		**40**		**786**	
	adenomas	13	43.3	8	17.4	15	33.3	9	24.3	0	0.0	58	43.0	13	7.6	103	40.1	8	20.0	227	28.9
	carcinomas	1	3.3	3	6.5	1	2.2	3	8.1	0	0.0	20	14.8	24	14.0	25	9.7	6	15.0	83	10.6
	neoplasia	14	46.7	11	23.9	16	35.6	12	32.4	10	41.7	78	57.8	37	21.5	128	49.8	14	35.0	320	40.7

† *All cases associated with neoplasia were S. bovis biotype I (listed under SGG), 2 SB cases had SB biotype II. Bold numbers represent total number of SBSEC cases per study (in columns) or total number of SBSEC cases per subspecies (in lines)*.

* *Studies with identification of SB subspecies*.

‡ *21 of 37 did not undergo colonoscopy, of 14 that underwent colonoscopy at index 7 had adenomas and 3 a carcinoma*.

*^n^ Devided into SB biotype I and II, biotype I is listed under SGG, 36 SB biotype II cases in total*.

*Untyped SBSEC cases are only included under “all SBSEC cases” and the total SBSEC species might thus not be equal to the sum of individual species cases*.

Interestingly, it is suggested that *SII* has a different association with neoplasia than *SGG*. *SGG* is more often associated with smaller asymptomatic non-advanced and advanced adenomas, while *SII* is generally an uncommon observation but more likely associated with larger tumors at a more advanced stage (Corredoira et al., 2013a; Stein, 2013). Based on this meta-analysis, this seems not the case as no carcinomas were found in any of the *SII* cases and adenomas and carcinomas were found almost equally in *SGP* (11.0 vs. 11.5%, respectively), although the association of *SGG* with adenomas was confirmed in five of seven studies (39.4 vs. 11.7% in carcinomas; Table 6) (Tripodi et al., 2004; Beck et al., 2008; Romero et al., 2011; Corredoira et al., 2013b, 2017).

Besides the obvious association with neoplasia, hepatobiliary disorders such as chronic liver diseases and liver cirrhosis seem to be highly prevalent among SBSEC IE patients (Gonzlez-Quintela et al., 2001; Tripodi et al., 2004). About 20% of all *SB* bacteremia cases have an association with underlying biliary origin of which 59% was isolated from polymicrobial infections, most frequently in combination with *Escherichia coli* and *Enterococcus* spp (Corredoira et al., 2014). The specific species associations with hepatobiliary disorders vary for *SGP* (10–83%) and *SL* (25–60%) followed by *SGG* (2–35%) and rarely for *SII, SGM* and *S. alactolyticus* (Beck et al., 2008; Romero et al., 2011; Lazarovitch et al., 2013; Corredoira et al., 2014; Sheng et al., 2014; Toepfner et al., 2014; Almeida et al., 2016). In rare cases, *SB* was obtained from cerebrospinal fluid and blood culture of patients with liver cirrhosis that developed bacterial meningitis (Barahona-Garrido et al., 2010). Urinary tract infections are a further infection caused by SBSEC members, particularly *SGP*, which are responsible for nearly 10% of cases in Vietnam (Poulsen et al., 2012).

### Immunogenic molecules and antibody response

#### Lessons from animal vaccination

SBSEC colonization and infection leads to immune responses by the host recognizing immunogenic molecules on SBSEC. Some of these immunogenic molecules have been discovered by the development of vaccines for *SB* in cattle. Parental immunization with *SB* using three boosters reduced clinical signs of lactic acidosis in cattle and has been a partly effective strategy. Feed intake in immunized sheep and steers was higher, there was a lower prevalence of diarrhea and they all survived grain challenge that results in the drop of rumen pH (Shu et al., 1999, 2000a; Gill et al., 2000). This immunization strategy resulted in a long-lasting serum and saliva anti-*SB* IgG response in steers and sheep. Although effective antibody levels were generated, it did not result in complete removal of *SB* from the rumen (Gill et al., 2000; Shu et al., 2000a,b, 2001). Feeding trials in mice using mozzarella cheese prepared from *SGM* or *SGM* in combinations with *S. thermophilus* and *Lactobacillus bulgaricus* as well as only *S. thermophilus* and *Lb. bulgaricus* also increased IL1-β levels for all combinations. However, only cheese prepared with *SGM* increased TNF-α and IL6 suggesting immune stimulation by *SGM* without inducing significant differences in mice body weight gain, spleen index and thymus index (Cho et al., 2012).

The bacteria cell surface likely plays a crucial role in SBSEC antigenicity. *SB* of CRC patients harbor an immunogenic surface molecule comparable to human choriogonadotropin. Vaccines prepared with chemically killed *SB* containing this human choriogonadotropin-like material on the cell surface have immunogenic properties and elicited antibody responses in rabbits. These antibodies also reacted with the human trophoblastic hormone and were similar to antibodies produced by human choriogonadotropin (Domingue et al., 1986; Acevedo et al., 1987). Furthermore, capsule and cell wall may contribute to the antigenicity of *SB*. This was observed via a lower cross-reactivity index for encapsulated strains (range 9.4–12.4%) than non-encapsulated strains (range 28.9–56.1%). This suggested antigenicity of capsule components and therefore a potential contribution of capsule and cell wall to the antigenicity of *SB*. In fact, colonization and antibody response of pigeons immunized with an *SGP* strain containing a thin irregular capsule (PDH 827) did not protect against a high-virulent strain *SGG* strain with a thick capsule (STR357). These findings indicate that supernatant proteins, glycans, fimbriae and the capsule may be involved in the induction of protective immunity against *SGG* infections but likely not across SBSEC species (Kane and Karakawa, 1969; Kane et al., 1972; Pazur and Forsberg, 1978; Pazur et al., 1978; Vanrobaeys et al., 1997; Kimpe et al., 2002).

#### Humoral reactions in IE

During establishment of IE, the immune system tries to clear the infection with *SB*. In several studies, the specific humoral response to *SB* has been investigated by crossing isolated pathogens or sterilized antigen compounds with the patients serum. By using *SGG* NCTC8133, 11 antigens between 41 and 130 kDa were detected via immunoblotting of two IE patient sera. Both IE patients produced strong IgG responses to many bands with a limited cross-reaction to enterococci (Burnie et al., 1987), but also considerable variation was found between *SE* and *SB* biotype II (*SII, SL*, or *SGP*) (Darjee and Gibb, 1993). The presence of antibody responses to common (c) antigen was specific to the *SB* isolates, but not to other Gram-positive IE isolates. This antigen did not lose its antigenicity by trypsin treatment or boiling, but changes in the pH from 7 to 5 had a significant reversible impact on antigenicity (Kaplan et al., 1983). In pigeons, a similar approach identified a 114-kDa immunogenic protein that was only recognized on highly virulent *SGGs* and two proteins (115 and 207 kDa) that were only recognized on low virulent *SGG* strains (Vanrobaeys et al., 2000b). Furthermore, antibodies to *SGG* are also common in the low molecular weight proteome. In the wall-extracted antigens, HlpA and RPL7/L12 have been identified as potential immunogenic proteins. These anchorless surface proteins might have important functions for host-microbe interactions as observed for the surface-exposed enolase from *Streptococcus pneumoniae* in tissue invasion (Tjalsma et al., 2007).

#### SBSEC humoral response for detection of CRC

Because of the specific antibody response to SBSEC and the strong correlation with colorectal adenomas and carcinomas, several studies have tried to identify adenoma and CRC patients utilizing the specific antibody response to SBSEC in general or to specific SBSEC surface proteins. The IgG antibody responses to whole cell extracted protein preparations from *SE* and *SB* were higher in patients with CRC than controls, but not for IgM titers (Darjee and Gibb, 1993). A similar approach with immunoblot of whole antigen extracts showed specific proteins at 22 kDa band that was associated with neoplasia (OR 7.98; 95% CI: 3.54–17.93) and in combination with a 30-kDa band resulted in an OR of 22.37 (CI 3.77–131.64) with a very high specificity (84.9% 22 kDa and 98.1% 30 kDa) and lower sensitivity (58.6% 22kDa and 30.1% 30 kDa) (Garza-González et al., 2012). These data were confirmed for wall-extracted antigen extracts from *SGG* showing increased seroprevalence in CRC and adenoma patients compared to colonoscopy controls (Abdulamir et al., 2009).

Specific approaches to antigenic proteins revealed that HlpA and RpL7/L12 wall-extracted antigen proteins from *SGG* were diagnostic for CRC (Tjalsma et al., 2006; Tjalsma, 2010). A detailed analysis revealed a positive correlation of anti-RpL7/L12 levels with age and the presence of colon polyps. Both adenoma and stage I/II CRC patients contained the highest anti-RpL7/L12 titers, which were significantly different from those of healthy individuals and advanced stage CRC patients, suggesting a temporal relation with early stage colonic lesions. A drawback of RpL7/L12 is the conserved nature within the bacterial kingdom and significant cross-reactivity resulting in significant overlap in serum from healthy controls and CRC patients (Boleij et al., 2010).

The IgG response to four *SGG* pili proteins (Pil1A, Pil1B, Pil2B, and Pil3B) was tested in single plex and multiplex assays. The IgG response to Pil1B was the best predictor for tumor presence, but did not result in the same response in all IE infected patients. CRC patients generally had response to only one of the four antigens with a maximum sensitivity of 20–43% by combination of four antigens (Boleij et al., 2012b). In the multiplex assay, the strongest association with CRC seropositivity was found by a combination of Pil1A and B (OR 3.54; 95% CI 1.49–8.44) and it was more predictive in patients with an age below 65 years. Although very specific, only a limited number of CRC cases could be identified using this approach (Butt et al., 2016). The association with Pil1B was confirmed in an independent cohort (OR 4.3; CI 2.14–8.65), but not for Pil1A. Interestingly, a specific association with non-advanced adenomas was found using a 6-marker panel with at least a positive seroresponse to two of six markers (OR 2.98; 95% CI: 1.18–7.57). With this marker panel, 27% of *SGG*-positives cases among non-advanced adenomas were identified compared to 11% of controls. Additionally, 7% of non-advanced adenomas were double-positive to Pil1A-Pil1B, compared to 0% of controls (*p*-value < 0.0001) (Butt et al., 2017). Data from a recent nested case-control study confirm previous observations that single marker panels are not sufficient to identify CRC patients and adenomas (Butt et al., 2017, 2018). This is potentially due to significant heterogeneity in IgG response between patients and depends on the antigenicity of the strain triggering the immune response. Nevertheless, these three studies confirm the strong and specific association of *SGG* with CRC and adenomas of the colon.

## Interpretation and conclusion

SBSEC pathobionts inhabit the GIT of animals and humans as well as food products featuring significant niche adaptation. While *SGG* retained most genetic and functional properties for a large variety of niches, *SGP, SL, SII* and particularly *SGM* have adapted more specifically to certain niches by gene gain and loss. SBSEC colonization as part of the rumen and gut microbiota is not ubiquitous and confers only a small sub-population of the overall microbiota during symbiosis. However, their ability to proliferate and colonize in dysbiosis, e.g., ruminal acidosis or in the tumor microenvironment is significantly enhanced and defines their pathogenicity.

Although SBSEC are not often found in association with tumors in microbiome studies, they likely have increased colonization levels at tumor sites. A potential tumor promoting ability of *SGG* may shape their microenvironment further and support the association with neoplastic sites. Increased immune responses in CRC patients toward *SGG* suggests a strong association with these sites. Considering the bacterial driver-passenger model, it seems that particularly *SGG* could be considered as a neoplastic site hijacker. *SGG* only excels when colonization factors and growth conditions in the environment are favorable. *SGG* is then able to further shape the microenvironment to its benefit.

This could also explain controversies between the relation of SBSEC with CRC in IE matched and unmatched patients. As SBSEC members are not strong ubiquitous gut colonizers and nonobligatory passengers on CRC, their prevalence among the general CRC population is low in contrast to patients with SBSEC IE. This also supports the proposed route of infection in humans and animals alike: SBSEC members, mostly *SGG*, are only able to colonize neoplastic tissues selectively. Subsequently, some strains have the capacity to translocate across the epithelial barrier into the blood system. Human blood-derived *SGG* possess the full *pil1, pil2*, and *pil3* loci, which enable adhesion to endothelial cells, exposed collagen and fibrinogen on heart valves and activate the human contact system. Via pili phase variation and expression of a capsular polysaccharide they can survive phagocytosis and minimize activation of the humoral immune response. *SGG* strains of animal origin and other SBSEC members without full pilus loci or different pilus proteins likely do not possess the same degree of infection ability and might depend on other factors to establish infection. Thus, animal and food-derived strains often adhere best to collagen type IV, whereas human-derived strains, particularly those of bacteremia or IE origin, predominantly show highest adhesion toward collagen type I. However, data is so far limited to presumed commensal animal strains whereas animal pathogens are not characterized leaving open question regarding virulence factors, host specificity but also zoonosis. In the light of SBSEC zoonotic potential, association with rural areas and prevalence in food, it will be relevant to determine the factors that enable colonization and establishment in animal and human hosts alike and provide comparison to food-derived strains. This will greatly contribute to a better understanding of transmission routes and development of intervention strategies to mitigate health risks associated with individual SBSEC members.

## Author contributions

CJ and AB equally shared all aspects of topic development, analysis, interpretation, manuscript writing and revision. Both authors have read and approved the submitted version of the manuscript. Both authors agree to be accountable for all aspects of the work in ensuring that questions related to the accuracy or integrity of any part of the work are appropriately investigated and resolved.

### Conflict of interest statement

The authors declare that the research was conducted in the absence of any commercial or financial relationships that could be construed as a potential conflict of interest.
